# Genomic and molecular characterisation of *Escherichia marmotae* from wild rodents in Qinghai-Tibet plateau as a potential pathogen

**DOI:** 10.1038/s41598-019-46831-3

**Published:** 2019-07-23

**Authors:** Sha Liu, Jie Feng, Ji Pu, Xuefang Xu, Shan Lu, Jing Yang, Yiting Wang, Dong Jin, Xiaochen Du, Xiangli Meng, Xia Luo, Hui Sun, Yanwen Xiong, Changyun Ye, Ruiting Lan, Jianguo Xu

**Affiliations:** 1 State Key Laboratory of Infectious Disease Prevention and Control, Collaborative Innovation Center of Diagnosis and Treatment of Infectious Diseases, National Institute of Communicable Disease Control and Prevention, Chinese Center of Disease Control and Prevention, Beijing, 102206 China; 2 Institute of Microbiology, Chinese Academy of Sciences, No. 1 Beichen West Road, Beijing, 100101 China; 3 School of Biotechnology and Biomolecular Sciences, University of New South Wales, Sydney, New South Wales Australia; 4 Department of Medical Microbiology and Parasitology, School of Basic Medical Sciences, Capital Medical University, Beijing, 100069 China; 5 Shanghai Institute of Emerging and Re-emerging infectious diseases, Shanghai Public Health Clinical Center, Shanghai, 201508 China

**Keywords:** Bacteriology, Bacterial genomics, Bacterial pathogenesis

## Abstract

Wildlife is a reservoir of emerging infectious diseases of humans and domestic animals. *Marmota himalayana* mainly resides 2800–4000 m above sea level in the Qinghai-Tibetan Plateau, and is the primary animal reservoir of plague pathogen *Yersinia pestis*. Recently we isolated a new species, *Escherichia marmotae* from the faeces of *M*. *himalayana*. In this study we characterised *E*. *marmotae* by genomic analysis and *in vitro* virulence testing to determine its potential as a human pathogen. We sequenced the genomes of the seven *E*. *marmotae* strains and found that they contained a plasmid that carried a *Shigella*-like type III secretion system (T3SS) and their effectors, and shared the same O antigen gene cluster as *Shigella dysenterae* 8 and *E. coli* O38. We also showed that *E*. *marmotae* was invasive to HEp-2 cells although it was much less invasive than *Shigella*. Thus *E*. *marmotae* is likely to be an invasive pathogen. However, *E*. *marmotae* has a truncated IpaA invasin, and lacks the environmental response regulator VirF and the IcsA-actin based intracellular motility, rendering it far less invasive in comparison to *Shigella*. *E*. *marmotae* also carried a diverse set of virulence factors in addition to the T3SS, including an IS1414 encoded enterotoxin gene *astA* with 37 copies, *E*. *coli* virulence genes *lifA/efa, cif*, and *epeA*, and the *sfp* gene cluster, *Yersinia* T3SS effector *yopJ*, one Type II secretion system and two Type VI secretion systems. Therefore, *E*. *marmotae* is a potential invasive pathogen.

## Introduction

Wildlife is a reservoir of emerging infectious diseases of humans and domestic animals^1^. These include both viral and bacterial pathogens. Wild waterfowl is the natural reservoir of all known subtypes of the influenza A virus, some of which contributes to the genesis of new subtypes to cause pandemics in humans^2^. Lyme disease caused by the bacterium *Borrelia burgdorferi* emerged from human contact with wild deer population in North America which carries the tick borne disease^3^. Proactive surveillance of wildlife for novel pathogens will help us to predict, prevent and manage emerging disease threats to humans.

Marmots are large terrestrial rodents widespread across much of northern Eurasia and North America^4^. *Marmota himalayana* mainly resides 2800–4000 m above sea level in the Qinghai-Tibetan Plateau, and is the primary animal reservoir of *Yersinia pestis*, the causative agent of bubonic plague^5^. Recently, we identified a number of novel bacterial species in wild Marmots^6–9^. With the expansion of human activity in the Qinghai-Tibetan Plateau, the chances of human–*M*. *himalayana* interaction have increased, which may allow transmission of pathogens from *M*. *himalayana* to humans. We recently analysed *E*. *coli* from faecal samples of *M*. *himalayana* and found multiple pathogenic types of *E*. *coli* present in these faecal samples^10^. We also isolated a new species, *Escherichia marmotae*, from the faeces of *M*. *himalayana*^11^.

In this study, we characterised *E*. *marmotae* by genomic analysis and *in vitro* virulence testing and found that it is a potential invasive pathogen with *Shigella*-like invasion genes. *Shigella* is a human only invasive pathogen and can invade intestinal epithelial cells, M cells, macrophages and interact with the intestinal mucosa, leading to bacillary dysentery^12^ *Shigella* carries an invasive plasmid, generally referred to as pINV which is also shared by enteroinvasive *E*. *coli* (EIEC)^13^, which encodes a type III secretion system (T3SS) for tissue invasion and other factors for its intracellular lifestyle^14,15^. For *Shigella*, the T3SS translocates a set of approximately 25 proteins from the bacterial cytoplasm directly into the eukaryotic host cell, where these “effector” proteins interfere with various host cell processes^14,15^. We found that *E*. *marmotae* from wild rodents carried a *Shigella*-like T3SS and associated effectors on a plasmid. The *E*. *marmotae* genome also contained a range of other virulence genes. Our results suggested that *E*. *marmotae* is a potential invasive pathogen.

## Results

### Genome sequencing of *E*. *marmotae*

**Figure 1:**
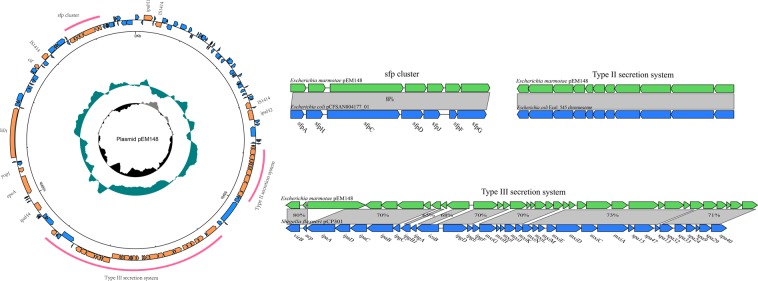
Circular representations of the pEM148 plasmid of *E*. *marmotae*. From the outside in (to scale): circle 1 represents genes on the positive and negative strands (scale is marked in 50 kb), circle 2 shows a plot of GC content (higher values outward), and circle 3 shows a plot of GC skew (G − C)/(G + C). The red curves indicate the region to be compared. Arrows of inset indicate predicted ORFs in both strands. Shown below gene bar are locus tags. Regions in light gray indicate homologous sequences and percentages of identity between two homologous genes at the nucleotide level. The inset depicts the comparisons of the plasmid regions of T3SS, T2SS and *sfp* gene cluster with the corresponding regions of pCP301 of *Shigella flexneri* str. 301 (NC_004851), *E*. *coli* 545 chromosome (NZ_CP018976) and *sfp* cluster of *E*. *coli* plasmid pCFSAN004177G_03(CP012494).

The seven‍ *E*. *marmotae* strains, HT073016, HT080709, HT080711, HT072503, HT073105, HT080118 and HT080401, isolated from the faeces of seven healthy *M*. *himalayana* in the Qinghai-Tibet plateau in China^11^, were sequenced using the Illumina Hi-Seq platform as draft genomes and the *E*. *marmotae* type strain HT073016 was further completely sequenced by Pacific Biosciences SMRT sequencing. The genome of HT073016 is 4.6 Mb with 50.65% G + C content and contains 4,438 genes. HT073016 also contained two plasmids named as pEM148 (Fig. 1) and pEM76 (Fig. S1). The size of pEM148 is 148,809 bp, encoding 145 genes and 2 tRNAs, with a G + C content of 44.49%. The size of pEM76 is 76,160 bp, encoding 75 genes, with a G + C content of 46.26% (Tables S1, S2). The G + C content of the plasmids is lower than that of the chromosome. Based on reads mapping, the other six *E*. *marmotae* strains also contained the two large plasmids present in HT073016.

### Phylogenetic relationship of *E*. *marmotae* to *E. coli/Shigella* based on the core genome

**Figure 2:**
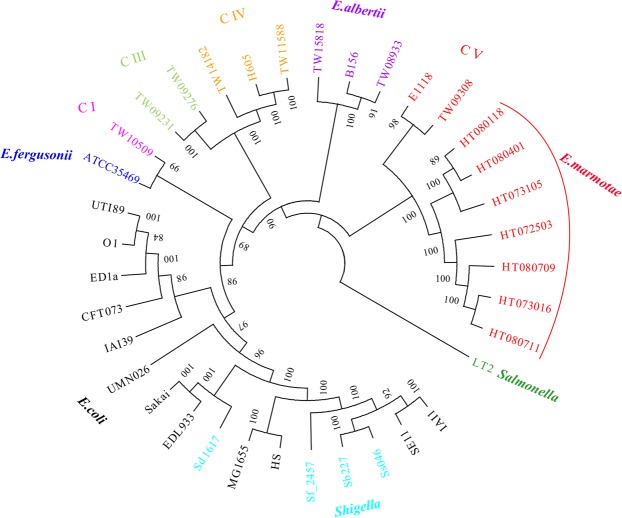
Phylogenetic tree of *E*. *marmotae* and 30 representative genomes. The tree was constructed using the maximum likelihood algorithms in Phylip based on the core genome SNPs. *Escherichia* clade I–V are marked as C I to C V. C V belonged to the species *E*. *marmotae*. Values on the branch are bootstrap values out of 100 from 1000 replicates. Species and strain names are colour coded. Note that *Shigella* strains belong to *E*. *coli*. *Salmonella* Typhimurium strain LT2 is used as an outgroup.

A total of 37 bacterial genomes including 9 *E*. *marmotae*, 12 *E*. *coli*, 3 *E*. *albertii*, 1 *E*. *fergusonii, 4 Shigella*, 6 *Escherichia* clade I–IV, and 1 *Salmonella* genomes, were used to constructed a phylogenetic tree using the core genome (Table 1, Fig. 2). A total of 123,829 genes were found among these genomes, of which, 2,053 single-copy orthologs were core genes using OrthoMCL (version 2.0)^16^. Consistent with our previous report^11^, the 9 *E*. *marmotae* strains including the 2 previously designated clade V strains and 7 strains isolated from the marmots were clustered together and well separated from *E*. *coli* and *Shigella*. The core genome tree confirmed that *E*. *marmotae* is a different species from *E*. *coli*/*Shigella*.

**Table 1:** Genomes list of phylogenetic analysis.

Strains	Lineage	Pathotype	Phylogeneticgroup	Host
MG1655	*E*. *coli*	Commensal	A	Human
HS	*E*. *coli*	Commensal	A	Human
SE11	*E*. *coli*	Commensal	B1	Human
IAI1	*E*. *coli*	Commensal	B1	Human
ED1a	*E*. *coli*	Commensal	B2	Human
Sakai	*E*. *coli*	EHEC	H	Human
EDL933	*E*. *coli*	EHEC	H	Food
UTI89	*E*. *coli*	UPEC	B2	Human
UPEC536	*E*. *coli*	UPEC	B2	Human
CFT073	*E*. *coli*	UPEC	B2	Human
APEC_O1	*E*. *coli*	APEC	B2	Chicken
UMN026	*E*. *coli*	UPEC	D	Human
IAI39	*E*. *coli*	UPEC	D	Human
TW10509	*Escherichia* clade I	Avirulent		Environment
TW09231	*Escherichia* clade III	Avirulent		Environment
TW09276	*Escherichia* clade III	Avirulent		Environment
H605	*Escherichia* clade IV	Avirulent		Environment
TW14182	*Escherichia* clade IV	Avirulent		Environment
TW11588	*Escherichia* clade IV	Avirulent		Environment
E1118	*Escherichia* clade V	Avirulent		Environment
TW09308	*Escherichi a*clade V	Avirulent		Environment
ATCC35469	*E*. *fergusonii*	Multiple		Human
TW08933	*E*. *albertii*	Serotype 7		Human
TW15818	*E*. *albertii*	Diarrheic		Human
B156	*E*. *albertii*	Avirulent		Human
301	*Shigella flexneri*	Diarrheic	S3	Human
Sd197	*Shigella dysenteriae*	Diarrheic	SD1	Human
Ss046	*Shigella sonnei*	Diarrheic	SS	Human
Sb227	*Shigella boydii*	Diarrheic	S1	Human
HT073016	*E*. *marmotae*	unknow		Marmot
HT080709	*E*. *marmotae*	unknow		Marmot
HT080711	*E*. *marmotae*	unknow		Marmot
HT072503	*E*. *marmotae*	unknow		Marmot
HT073105	*E*.*marmotae*	unknow		Marmot
HT080118	*E*. *marmotae*	unknow		Marmot
HT080401	*E*. *marmotae*	unknow		Marmot
LT2	*Salmonella* Typhimurium	Typhimurium	outgroup	Human

### The *E*. *marmotae* genome contains a *Shigella*-like type III secretion system and other *Shigella* virulence gene homologues

The plasmid pEM148 was found to contain a region homologous to the invasion region of the invasion plasmid pCP301 (pINV) of *Shigella flexneri* strain 301. The region contained 34 ORFs that share 45% to 94% identity with the type III secretion system (T3SS) of pCP301 (Fig. 1, Table S3). The genetic organization of the T3SS gene cluster of HT073016 is co-linear with that of *Shigella* pCP301 for nearly the entire gene cluster from *virB* to *spa40* including the T3SS effectors for invasion and intracellular survival, assembly and function of the T3SS, and molecular chaperones (Fig. 1).

However, some genes are missing or damaged. In *Shigella*, *ipaA*, encoding an invasin, is located between *acp* and *ipaD*. In HT073016, the *ipaA* is truncated (1,119 bp) at the 3′ end, covering only 59% of the full length *ipaA* (1,902 bp), which has likely become a pseudogene. The truncated *ipaA* is fused with an unknown sequence to form a large mosaic gene of 4326 bp. The non-*ipaA* sequence shared little homology with known genes in GenBank by BLAST. Deletion of *ipaA* caused an ∼1000 fold decrease in the ability of *S*. *flexneri* to invade HeLa cells^17^. *ipaJ* encoding a cysteine protease that demyristoylate host proteins^18^ and located at the 5′ end of the *Shigella* invasion gene cluster is missing in HT073016. *virF*, encoding a master regulator of T3SS^19^, is also missing in *E*. *marmotae*. Another *Shigella* virulence gene, *icsA* (also known as *virG*), which is encoded on the *Shigella* pCP301 plasmid but outside the invasion region, was also missing in HT073016. IcsA mediates actin based motility inside the host cell in *Shigella*^20^.

*Shigella* and EIEC characteristically carry multiple copies of *ipaH* which encode a novel class of E3 ubiquitin ligase secreted via the T3SS^21,22^. We found 11 *ipaH*-like genes and named them as *ipaH1* to *ipaH11*: Four on plasmid pEM148, five on plasmid pEM76, and two on the chromosome (Table S1). Seven shared homology with *Shigella ipaH* (Table S1), among which *ipaH8* on plasmid pEM76 shared 92% identity with *ipaH9*.8 on *Shigella* pCP301 with 99% coverage. The *ospC* found on pEM76 shared 79% nucleotide identity with *ospC4* on *Shigella* pCP301.

### *E*. *marmotae* is capable of invading epithelial cells *in vitro*

**Figure 3:**
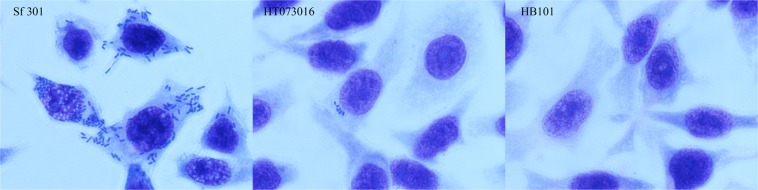
Invasion of epithelial cells by *E*. *marmotae in vitro*. HEp-2 cell invasion by *E*. *marmotae* HT073016, *S*. *flexneri* str. 301 (positive control) and *E*. *coli* HB101 (negative control) as labelled. Infected HEp-2 cells were fixed by methanol and then stained with Geimsa.

Cell invasion is a key characteristic of *Shigella* pathogenicity. The ability to invade epithelial cells by *E*. *marmotae* strain HT073016 was evaluated using the cell culture invasion assay, which is well established assays for *Shigella*^23,24^. In the HEp-2 cell culture invasion assay, the average number of bacteria entered into HEp-2 cells was 5 bacteria per HEp-2 cell for HT073016, in contrast, there were an average of 200 bacteria per HEp-2 cell for *S*. *flexneri* str. 301 (Fig. 3). We further quantitated the number of intracellular bacteria by colony forming unit (CFU) count. Extracellular bacteria were removed by gentamicin and intracellular bacteria were released by lysing the HEp-2 cells. The number of CFUs for *E*. *marmotae* HT073016 and *S*. *flexneri* str. 301 was 46 ± 16 and 52,000 ± 270 respectively, suggesting that there is >1000 times difference between *E*. *Marmotae* and *Shigella* in invasion ability. We used *E*. *coli* HB101 as negative control and no intracellular bacteria were observed by microscopy and CFU count (Fig. 3). These results indicate that *E*. *marmotae* has ability to invade epithelial cells, but its invasion ability is much lower than *Shigella*.

### *Shigella* regulator *virF* can control *E*. *marmotae* T3SS to be responsive to temperature

**Figure 4:**
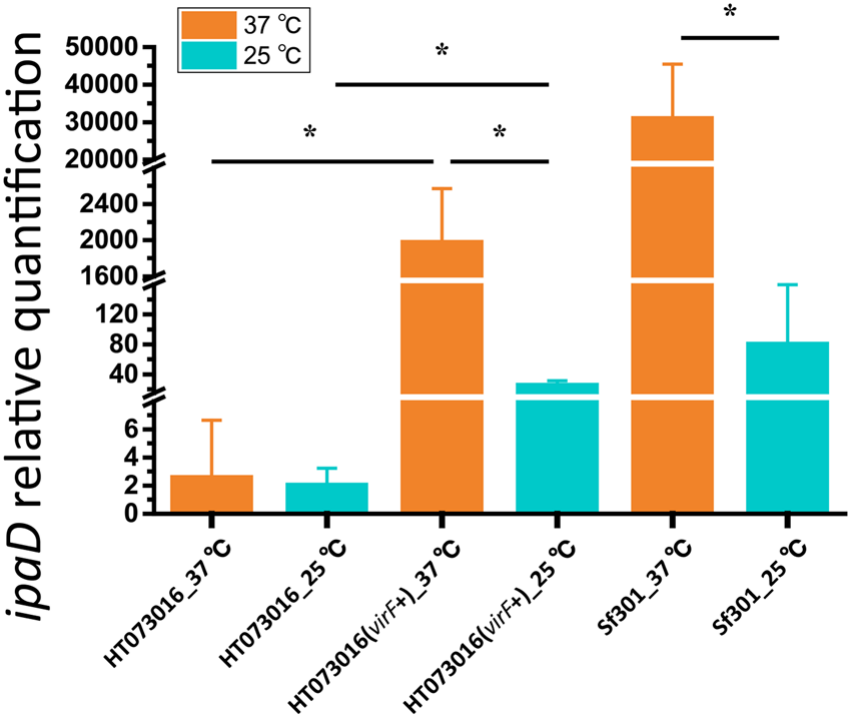
Expression of *ipaD* under different temperature conditions as determined by qRT-PCR. Total RNA was harvested from HT073016, HT073016(*virF*+) and *S*. *flexneri* strain 301 cultivated at 25 °C and 37 °C. Relative transcript levels were calculated using the ∆∆CT method and fold changes in comparison to HT073016 at 37 °C. All values have been normalized to the endogenous reference gene *recA*. Means and standard deviations stand for three independent experiments were shown with * being p < 0.05 in the indicated comparisons. Error bars are +SD.

The major trigger to induce the expression of the virulence genes of the *Shigella* invasion plasmid is a temperature shift to 37 °C before *Shigella* enters the host^25^. *virF*, as a global positive regulator, is responsible for triggering T3SS expression in *Shigella*^26^. However, no *virF* homologue was found in *E*. *marmotae* HT073016. We examined the response of T3SS of *E*. *marmotae* HT073016 to temperature shift by measuring T3SS *ipaD* expression. The *recA* gene was used as a reference. The *ipaD* expression of *E*. *marmotae* HT073016 at 25 °C and 37 °C was almost the same and was considerably lower than *S*. *flexneri* str. 301 at 25 °C (Fig. 4). In contrast, the *ipaD* expression of *S*. *flexneri* str. 301 was responsive to temperature shift with 382 times higher expression at 37 °C than 25 °C. Therefore, unlike *Shigella*, the expression of T3SS of *E*. *marmotae* HT073016 is not responsive to temperature modulation. We then cloned the *virF* gene from *S*. *flexneri* str. 301 with its promoter region into HT073016 to determine whether *Shigella virF* can regulate *E*. *marmotae* T3SS expression. The *ipaD* gene expression of recombinant HT073016 (*virF*^+^) was increased by 73 folds, when the temperature shifted from 25 °C to 37 °C (Fig. 4), demonstrating that *Shigella virF* can exert control on the expression of *E*. *marmotae* T3SS. We further tested the recombinant HT073016 (*virF*^+^) for its invasion ability (Fig. S2). The number of intracellular bacteria (CFUs) for the *virF* transformant was 198 ± 66 in comparison to the CFU of 46 ± 16 for the wild type. The difference is statistically significant (t test, p < 0.01).

### The *E*. *marmotae* genome contained virulence genes of other enteric bacteria

There are four singularly located virulence genes found in pEM148: An enterohaemorrhagic *E*. *coli* (EHEC) *epeA, E*. *coli lifA/efa* (lymphocyte inhibitory factor A), *cif* (cycle inhibiting factor) and *Yersinia yopJ* homologue. *epeA* encodes a serine protease belonging to the family of serine protease autotransporters of *Enterobacteriaceae* (SPATEs)^27^. *Shigella sepA* also belongs to SPATES family^27^ and is absent in *E*. *marmotae*. *E*. *marmotae lifA/efa* shares 80% identity with *E*. *coli lifA/efa* which has a dual role in suppressing cytokine expression and functioning as an adhesin^28,29^. The *E*. *marmotae cif* shares 74% identity with *E*. *coli cif* which encodes a T3SS effector that induces host cell cycle arrest and reorganization of the actin cytoskeleton^30^. The *E*. *marmotae yopJ* shares 82% identity with *Yersinia yopJ*, which encodes an acetyl transferase targeting and inhibiting host immune cells^31^ (Table S4).

There are four gene clusters associated with virulence. A type II secretion system (T2SS) is present on plasmid pEM148 which shares an overall 91% identity with those of *E*. *coli* and *Shigella* (Fig. 1). However, the *E*. *coli* and *Shigella* T2SS was located on the chromosome^32^. A sorbitol-fermenting fimbriae protein (Sfp) locus is present on pEM148 which contained seven genes and shared 88% identity with that of *E*. *coli* (Fig. 1). The *sfp* locus is associated with adhesion to host cells in EHEC^33^. In addition to plasmid encoded virulence genes, HT073016 contains 2 type VI secretion systems (T6SS) on the chromosome (Table S5), one of which, T6SS1, is more homologous to that of *Salmonella*, while the other (T6SS2) is more similar to the one in *E*. *coli* O42 (Fig. S3). However, T6SS2 appears to be partial as the majority of the *E. coli* T6SS homologs were absent (Table S5).

### The *E*. *marmatae* genome contained numerous copies of IS1414 that encodes an enterotoxin

The *E*. *marmotae* genome also contained a large number of ISs (121) (Table 1). The predominant species are IS1414 (37), IS4 (36), followed by IS*Cro1* (24) and IS*Ec16* (19). IS1414 is found on both the chromosome (32 copies) and the two plasmids (5 copies) (Tables 2, S6). Interestingly IS1414 carries a heat-stable enterotoxin gene *astA* inside the IS as previously found in *E*. *coli*^34,35^. IS1414 is found at a much smaller number from 1 to 8 copies in *E*. *coli*. No IS1414 was found in the *S*. *flexneri* str. 301 genome.

**Table 2:** IS distribution in the genome of HT073016.

Name	Length(bp)	IS family	HT073016	pEM148	pEM76
IS4	1426	IS4	33	1	2
IS*1414*	1314	IS256	32	3	2
ISCro1	2699	IS66	19	2	3
ISEc16	1244	IS3	14	3	2
IS1H	764	IS1	1	2	0
ISEc1	1291	ISAs1	1	0	0
ISEc13	1550	IS4	1	0	0
IS911	1250	IS3	0	0	1
Total			101	11	10

### *E*. *marmotae* carries the same O antigen gene cluster as *Shigella dysenteriae* type 8 and *E coli* O38

All seven *E*. *marmotae* strains carry the same O antigen. The *E*. *marmotae* O-antigen biosynthesis gene cluster shares 99.41% identity with that of *E*. *coli* O38 and 99.24% with that of *Shigella dysenteria* type 8 (SD8) which is much higher than chromosomal genes between the HT073016 and *Shigella* at around 60%, suggesting that one or all acquired the O antigen gene cluster from the same source recently (Fig. S4). Slide agglutination test confirmed that *E*. *marmotae* HT073016 agglutinated with SD8 diagnostic antiserum for *Shigella* serotyping.

## Discussion

*Shigella* evolved from commensal *E*. *coli* through acquisition of key virulence genes primarily carried by the invasion plasmid pINV which is shared by *Shigella* and EIEC^13^. There had been no reports of any other species carrying a similar plasmid. In this study we show that *E*. *marmotae* strains isolated from wild rodents from the Qinghai-Tibet plateau contained a plasmid that carried a T3SS gene cluster other virulence genes with high homology to that on the pINV, and also virulence genes on the chromosome. *E*. *marmotae* was also found to be invasive to HEp-2 cells. Thus, *E*. *marmotae* is a potential invasive pathogen. *E*. *marmotae* also shared the multicopy *ipaH* gene with *Shigella* and EIEC, which participates to modulate the immune response of the host^36^.

*Shigella* and EIEC arose within *E*. *coli* multiple times independently by gaining a similar invasion plasmid^37^. However, no *Shigella*-like plasmid or *Shigella*-like T3SS system in other species had been reported previously. The finding that *E*. *marmotae* gained a similar T3SS and other invasion related genes showed parallel evolution of the *Shigella*-like T3SS and associated virulence genes within the genus *Escherichia*. Firstly, the T3SS gene cluster showed co-linearity over the entire gene cluster between *E*. *marmotae* and *Shigella* and both T3SS were carried by a plasmid, although the two plasmids shared little similarity in other regions. Secondly, most of the key *Shigella* T3SS effectors secreted are present in *E*. *marmotae*.

Cell invasion experiments showed that *E*. *marmotae* was much less invasive than *Shigella*. The absence of three important pathogenic mechanisms: temperature modulation and IcsA-based intracellular motility and absence of ipaA may have contributed to this difference. Therefore *E*. *marmotae* is a less invasive pathogen. The master regulator VirF is absent in *E*. *marmotae*. *VirF* responds to a diverse range of environmental signals^26^. Our experiments showed that introduction of *Shigella virF* with its promoter region into *E*. *marmotae* rendered the *E*. *marmotae* T3SS gene expression responsive to temperature because the *virF* gene from *Shigella* contain its promoter region which responds to temperature and also increased the invasiveness to HEp-2 cells. *E*. *marmotae* lacked IcsA for actin based motility which is used for movement within and between epithelial cells. For *Shigella*, the key virulence factors were all encoded on pINV. It has been shown that pINV is a composite plasmid originated from different sources^14^. For *E*. *marmotae*, the plasmid pEM148 is also a composite plasmid with G + C content of the invasion region (T3SS gene cluster) differed from the other regions of the plasmid. *E*. *marmotae* is yet to acquire other key virulence genes such as *icsA* to develop full blown *Shigella-*like pathogenicity.

In addition to the virulence genes similar to those of *Shigella*, the *E*. *marmotae* genome also contained other virulence genes. These include *E*. *coli* homologues, *lifA/efa*, *astA*, *cif*, and the *sfp* gene cluster, *Yersinia yopJ*, one T2SS, and two T6SSs. It is particularly interesting that the *astA* gene which is embedded entirely within IS1414 is present in *E*. *marmotae* with 37 copies. *astA* encodes heat-stable enterotoxin 1 (EAST1) which was initially discovered in *enteroaggregative E*. *coli*^34^. Based on the predominance of IS1414 in *E*. *marmotae* and its low frequency in *E*. *coli*, it is highly likely that the *E*. *coli* IS1414 and its associated *astA* gene was originated from *E*. *marmotae*. *Escherichia* Clade V strain TW09308, genetically a member of *E*. *marmotae*^11^, also contained IS1414 and the *astA* gene^38^. *Escherichia* Clade V strains have been found in the environment including fresh water and marine sediments and from different geographical regions including Australia, the US and Italy^38^. The environmental *Escherichia* Clade V strains from the marine sediments were shown to carry the *astA* gene and were also found to be adhesive to human epithelial cells^39^. Therefore *E*. *marmotae* may contain both widely distributed environmental strains (ie. *Escherichia* Clade V^36^) and animal intestinal strains such as those isolated from *M*. *himalayana* with differences in pathogenicity. *E*. *marmotae* is likely to be an environmental organism turning into a pathogen.

However, the 7 *E*. *marmotae* strains we isolated were from apparently healthy marmots. It remains to be demonstrated whether *E*. *marmotae* can cause disease in humans or other animals. The genome content of virulence genes and our cell invasion experiments suggest that *E*. *marmotae* is likely to be an invasive pathogen. Further studies will be required to demonstrate its pathogenicity and disease-causing potential to humans.

This study further supports that wild marmots are a reservoir of potential human pathogens and virulence genes. We previously sampled *E*. *coli* from *M*. *himalayana* and the majority of the 112 *E*. *coli* strains sequenced carried multiple virulence genes and in particular they carried virulence genes from different pathogenic types of *E*. *coli* as potential hybrid pathogens^10^. Qinghai-Tibet plateau is a relatively pristine environment. The area is sparsely populated and human activity is low, due to its high altitude (2,700 to 5,450 meters above sea level) and harsh climate in winter which make it less suitable for human inhabitation. There are many wild animal species in the Qinghai-Tibet plateau. Therefore, it is likely that other wild animals also carry *E*. *marmotae* and other known or unknown pathogens.

In conclusion, we characterised *E*. *marmotae* strains isolated from wild marmots and showed that *E*. *marmotae* acquired a plasmid that carries a *Shigella*-like T3SS system. It also carried many homologues of *Shigella* effectors. However, cell invasion assays showed that *E*. *marmotae* is far less invasive than *Shigella*, which may be due to that *E*. *marmotae* lacks the VirF mediated regulatory system to be responsive to environmental changes and the *Shigella* IcsA-actin based intracellular motility. This study also provides further support that wild animals are reservoirs of potential novel human pathogens.

## Materials and Methods

### Bacteria strains, cell culture and media

*E*. *coli* HB101, *S*. *flexneri* str. 301, *E*. *marmotae* strains HT073016, HT080709, HT080711, HT072503, HT073105, HT080118 and HT080401 were grown in Luria-Bertani (LB) broth or on LB agar at 37 °C. HEp-2 cells were cultured in DMEM (Gibco) with 10% bovine fetal calf serum.

### Genome sequencing, annotation and whole genome phylogenetic analysis

DNA was prepared with the Wizard Genomic DNA Purification Kit (Promega, USA). Seven isolates were sequenced using Illumina HiSeq2000 by Beijing Genomics Institute. HT073016 was further sequenced using Pacific Biosciences RSII DNA sequencing system (Pacific Biosciences, Menlo Park, CA, USA) by Tianjin Biochip Corporation. De novo assembly of the insert reads of Pacific Biosciences SMRT sequencing was performed with the Hierarchical Genome Assembly Process (HGAP_Assembly.2) algorithm in SMRT Portal (version 2.3.0). Circularization was achieved by manual comparison and removal of a region of overlap, and the final genome was confirmed by remapping of sequence data. Initial annotation of the genome was done using the Rapid Annotation using Subsystem Technology online interface, and further annotated by BLASTP and BLASTN against NCBI’s conserved domain database and non-redundant databases. Virulence gene annotations were recovered using VFDB (http://www.mgc.ac.cn/VFs/download.htm). Plasmids carrying virulence genes were then selected for a more in-depth annotation. Each CDS initially annotated as transposase was further annotated by BLASTN against the ISFINDER database. Insertion sequence terminal inverted repeats (TIR) and direct repeats (DR) were identified using comparisons with known published elements. Schematic map of plasmids and gene organization diagrams were drawn with in-house Perl scripts and Inkscape^40^. The phylogenetic tree based on the core genome SNPs was constructed using the maximum likelihood algorithms in PHYLIP (http://evolution.gs.washington.edu/phylip.html). Bootstraps were performed with 1,000 replicates. The resulting phylogeny was displayed by SplitsTree (http://en.bio-soft.net/tree/SplitsTree.html), and edited by iTOL and Adobe illustrator.

GenBank accession number of genomes sequenced in this work is SRS2488458 - SRS2488464 (Bioproject identification number PRJNA401298), CP025979- CP025981.

### Epithelial cell invasion assay

HEp-2 cells were seeded at 1 × 10^5^ cells/well into 24-well cell culture plates (Costar, Corning) and cultured overnight. Cells were washed three times with pre-warmed DMEM to remove the antibiotics and serum before addition of bacteria. Subconfluent monolayers of HEp-2 cells were infected with approximately 2 × 10^6^ exponential-phase bacteria in DMEM at 37 °C. Following an initial invasion period of 1 h, cells were washed three times by DMEM, and the infection was allowed to continue for an additional 1 h after the addition of 100 μg/ml gentamicin, which kills extracellular but not intracellular bacteria. The infected HEp-2 cells were washed, followed by fixation with methanol and then stained with Giemsa for analysis under a light microscope (Nikon ECLIPSE 80i). The invasive capacity of bacteria was further measured by counting the viable number of internalized bacteria using colony forming unit (CFU) count method on LB agar plate after the cells were lysed by 0.25% Triton X-100 to release the bacteria. The *S*. *flexneri* str. 301, and *E*. *coli* HB101 were used as positive and negative control, respectively.

### Recombinant strain construction

First, we amplified the DNA region containing *S*. *flexneri* str. 301 *virF* gene and its promoter using the primers (*virF*-promoter-F: 5′-AGAAGCTGCATAAGCTCTTTCTTC-3′; *virF*-promoter-R: 5′-GGGGAAAACCCATCTGGCAA-3′). The PCR product was purified by Gel Extraction Kit (Qiagen). Thereafter the purified product was cloned into pMD18-T vector (Takara) and transformed into *E*. *coli* JM109 for amplification. Eventually after extracted by Plasmid Mini Kit (Omega), the recombinant plasmid was transformed into competent *E*. *marmotae* HT073016 cell to construct *E*. *marmotae* HT073016(*virF*^+^).

### Quantitative Real-time PCR assay

*E*. *marmotae* HT073016, HT073016(*virF*^+^) and *S*. *flexneri* str. 301 were cultured in the LB medium at 37 °C and 25 °C with constant shaking until reached the exponential phase of growth. Then the total RNA of each sample was extracted by using the RNeasy Mini kit (Qiagen) and digested by TURBO DNase (Ambion) and reversely transcribed to cDNA by PrimeScript™ RT Master Mix (Takara). RNA concentrations were determined by a NanoDrop spectrophotometer. Transcriptional levels of the *ipaD* gene from *E*. *marmotae* HT073016, HT073016(*virF*^+^) and *S*. *flexneri*str. 301 were measured by the quantitative real-time PCR. Primers for *ipaD* and reference gene *recA* were listed as followed (*ipaD*-F: 5′-GATAATGCAAAATATCAGGCATGGAA-3′, *ipaD*-R: 5′-CATGAGCTTATTGTACTACTCAAAACCTT-3′; *recA*-F: 5′-ACAAACAGAAAGCGTTGGCG-3′, *recA*-R: 5′-CCAAGCGCGATATCCAGTGA-3′). Reactions were prepared using SYBR Premix Ex Taq^TM^ II (Takara) in a total volume of 25 μL. The real-time PCR assays were performed by using Rotor-Gene Q (Qiagen) following the amplification program: 45 cycles at 95 °C for 5 sec, 60 °C for 30 sec and 72 °C for 30 sec. For each sample, the raw real-time PCR data for the target gene *ipaD* were normalized against the reference gene *recA*, and fold changes were calculated using the △△*C*_T_ method as reported by Livak and Schmittgen^41^. The results were based on three individual experiments.

## Supplementary information

Genomic and molecular characterisation of Escherichia marmotae from wild rodents in Qinghai-Tibet plateau as a potential pathogen
