# Supplementary material for: Genomic and molecular characterisation of Escherichia marmotae from wild rodents in Qinghai-Tibet plateau as a potential pathogen
Source: Sci Rep. 2019 Jul 23;9:10619. doi: 10.1038/s41598-019-46831-3 (PMC6650469; doi:10.1038/s41598-019-46831-3)
Supplement: Supplementary file 1 — Genomic and molecular characterisation of Escherichia marmotae from wild rodents in Qinghai-Tibet plateau as a potential pathogen [file 41598_2019_46831_MOESM1_ESM.pdf]

Genomic and molecular characterisation of *Escherichia marmotae* from wild rodents in Qinghai-Tibet plateau as a potential pathogen

Sha Liu[1,4,#], Jie Feng[2,#], Ji Pu[1,#], Xuefang Xu[1,#], Shan Lu[1,#], Jing Yang[1], Yiting Wang[1], Dong Jin[1], Xiaochen Du[2], Xiangli Meng[1], Xia Luo[1], Hui Sun[1], Yanwen Xiong[1], Changyun Ye[1], Ruiting Lan[3,*] and Jianguo Xu[1,5,*]

1 State Key Laboratory of Infectious Disease Prevention and Control, Collaborative Innovation Center of Diagnosis and Treatment of Infectious Diseases, National Institute of Communicable Disease Control and Prevention，Chinese Center of Disease Control and Prevention, Beijing 102206, China

2 Institute of Microbiology, Chinese Academy of Sciences, No.1 Beichen West Road, Beijing 100101, China

3 School of Biotechnology and Biomolecular Sciences, University of New South Wales, Sydney, New South Wales, Australia

4 Department of Medical Microbiology and Parasitology, School of Basic Medical Sciences, Capital Medical University, Beijing 100069, China

5 Shanghai Institute of Emerging and Re-emerging infectious diseases, Shanghai Public Health Clinical Center, 201508, China.

*: Corresponding authors

Prof Jianguo Xu, xujianguo1@icdc.cn; Prof Ruiting Lan, r.lan@unsw.edu.au

#: those authors contributed equally

Supplemental Material

A

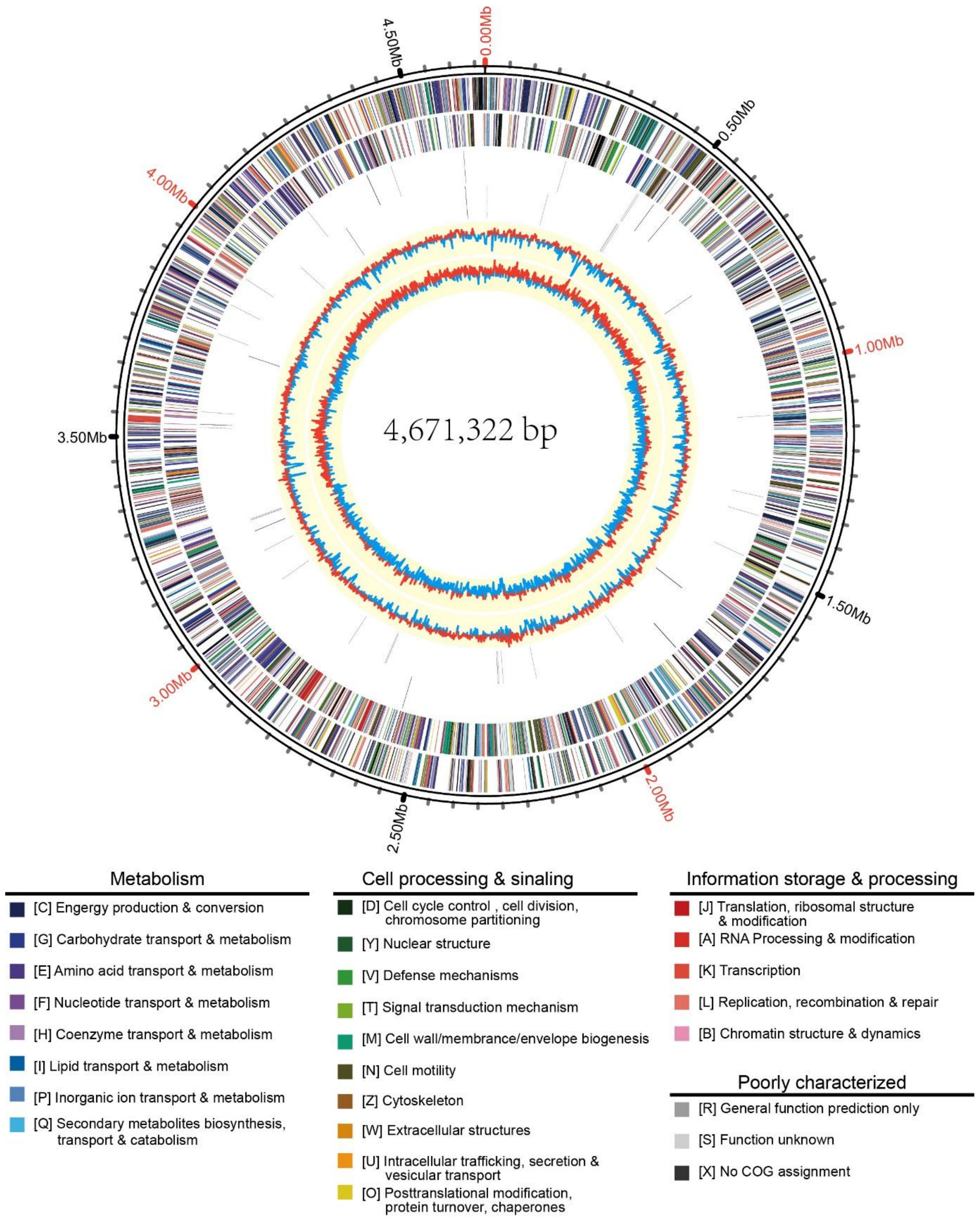

B

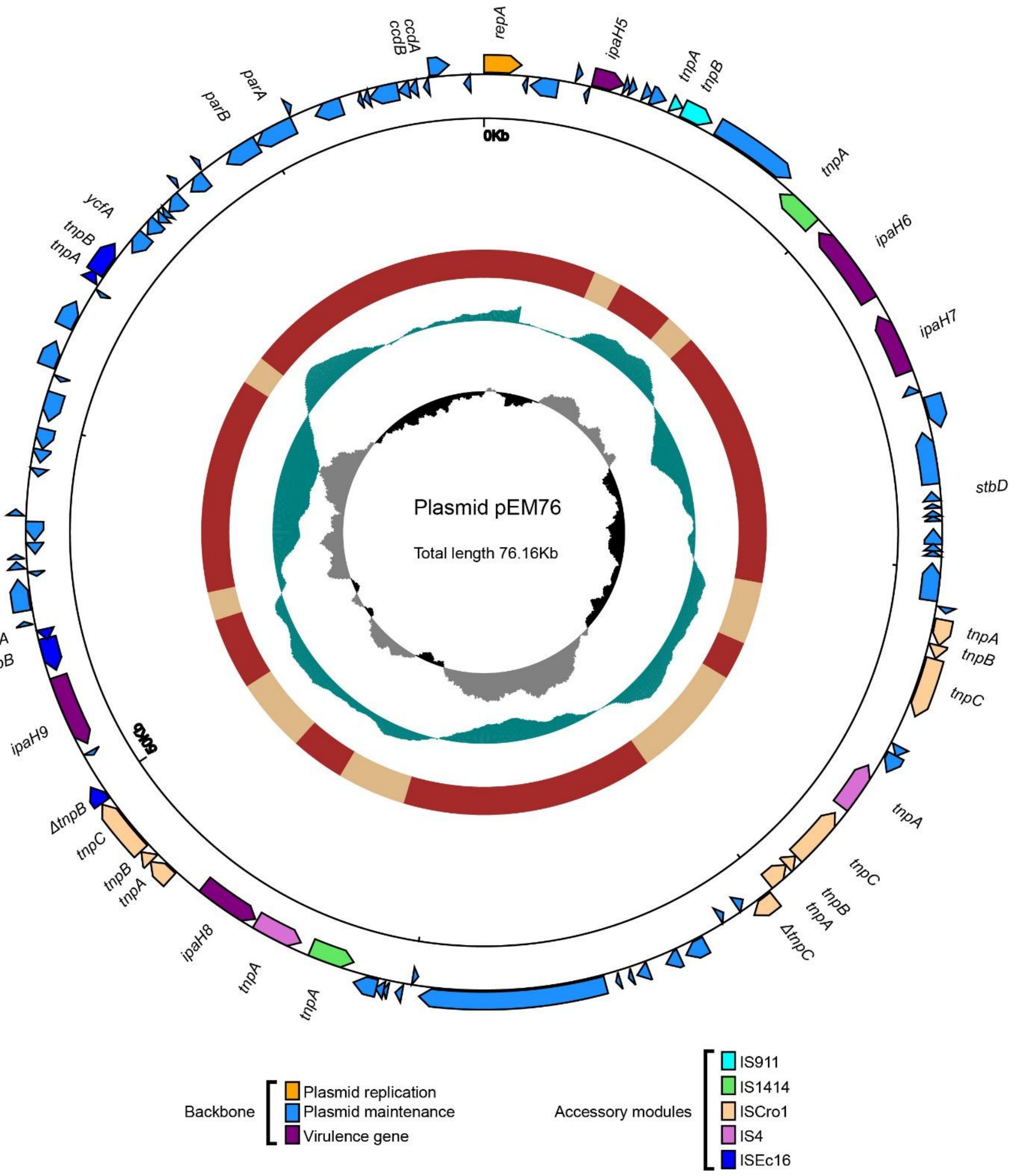

Fig S1 Circular representation of the HT073016 chromosome and plasmid pEM76

(A) Circular representation of the HT073016 chromosome. Circles range from 1 (outer circle) to 7 (inner circle). Circle 1, scale marks of the genome (0.5Mbp), Circles 2 and 3, protein-coding genes on the forward strand and reverse strand respectively. Circles 4 and 5, tRNA

(black) and rRNA (red) genes on the forward strand and reverse strand respectively. Circle 6, G + C content with a window size of 10 kb. Circle 7, GC skew (G ± C/G + C). Protein-coding genes are color coded according to their COG categories.

(B) Circular representation of plasmid pEM76. From the outside in: circle 1 represents genes on the positive and negative strands (scale is marked in 50 kb), circle 2 shows a plot of GC content (higher values outward), circle 3 shows a plot of GC skew (G-C)/(G+C), respectively.

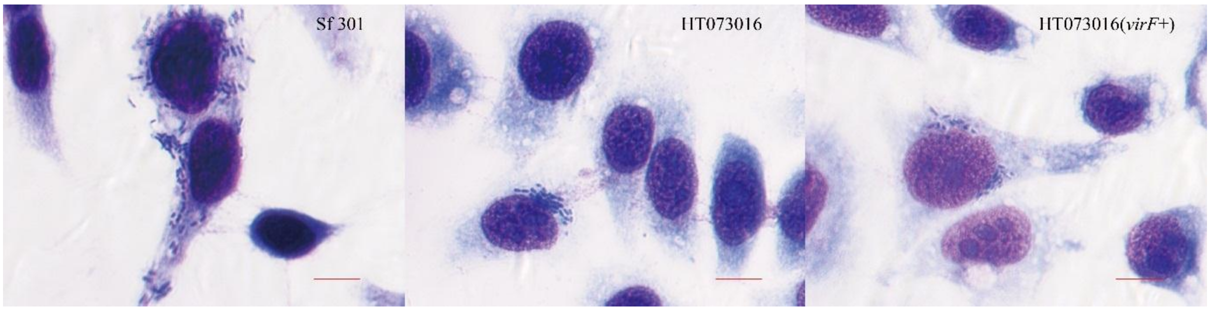

Fig S2 Invasion of epithelial cells by *E. marmotae* and *E. marmotae* (*virF*+) *in vitro.* HEp-2 cell invasion by *E. marmotae* HT073016, *E. marmotae* (*virF+*), *S. flexneri* str. 301 (positive control) and *E. c.oli* HB101 (negative control, not showed) as labelled. Infected HEp-2 cells were fixed by methanol and then stained with Geimsa.

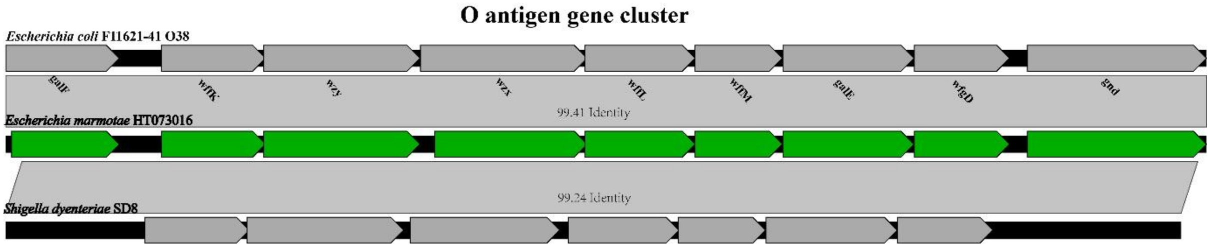

Fig S3 Graphic representation of O-antigen gene cluster of *E. marmotae* HT073016 in comparison with the related strains: type 38 O-antigen of. *E. coli* F11621 (top) and type 8

O-antigen of *S. dysenteriae* (below)

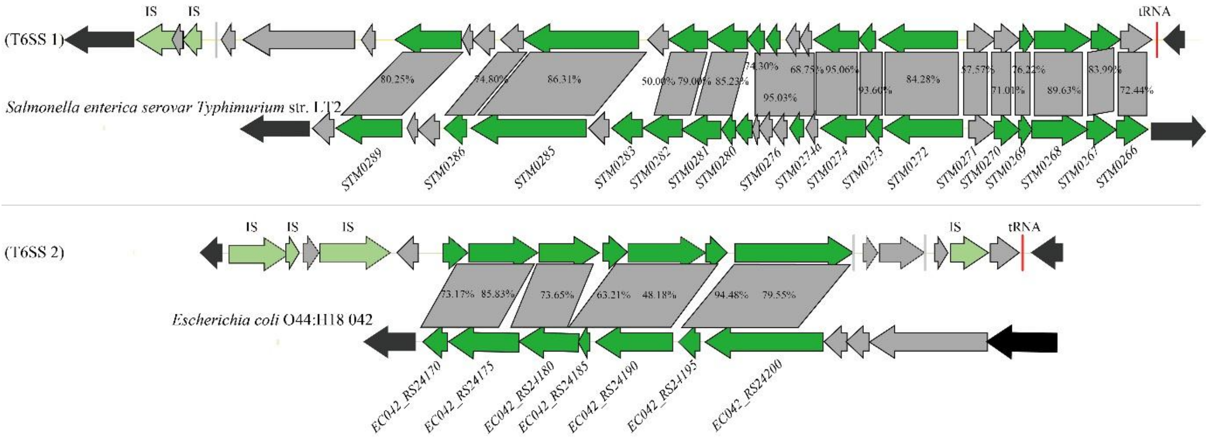

Fig S4 Diagram showing the genetic organization of the type VI secretion system (T6SS) gene cluster of *E. marmotae* HT073016 compared to the T6SS clusters of *Salmonella enterica serovar* Typhimurium str. LT2 and enteroaggregative *E. coli* (EAEC) strain O42. Shown in green are the genes of T6SS of *E. marmotae* HT073016. Genes in gray color are hypothetical protein. Genes in dark green color are transposase. Genes in blue color are T6SS of the other strains. Genes in black exhibit other functional genes. Regions in light gray indicate homologous sequences in % identity between two homologous genes at the nucleotide level.

Table S1 The gene lists of plasmid pEM148 in *E. marmotae* HT073016

	start	stop	strand	locus-tag	product
CDS	1	696	+	C1192_23870	RepB family plasmid replication initiator
CDS	1648	3330	+	C1192_23875	E3 ubiquitin--protein ligase
CDS	3642	3958	+	C1192_23880	IS3 family transposase
CDS	3997	5205	-	C1192_23885	IS256 family transposase
CDS	5279	6115	+	C1192_23890	IS3 family transposase
CDS	6330	7319	-	C1192_23895	non-LEE encoded effector protein NleB
CDS	7324	7503	-	C1192_23900	hypothetical protein
CDS	7558	8352	+	C1192_23905	ribonuclease
CDS	8966	10150	+	C1192_23910	cysteine protease
CDS	10747	11274	+	C1192_23915	hypothetical protein
CDS	11271	11878	-	C1192_23920	hypothetical protein
CDS	12871	13380	+	C1192_23925	DUF2726 domain-containing protein
CDS	13590	14426	+	C1192_23930	OspB
CDS	14648	15444	-	C1192_23935	IS3 family transposase
CDS	16232	16483	+	C1192_23940	hypothetical protein
CDS	16606	17058	-	C1192_23945	lytic transglycosylase
CDS	17550	17816	+	C1192_23950	hypothetical protein
CDS	17813	18963	-	C1192_23955	IS3 family transposase
CDS	19390	19962	-	C1192_23960	type III effector
CDS	20159	21309	+	C1192_23965	IS3 family transposase
CDS	21610	22158	+	C1192_23970	IS630 family transposase
CDS	22249	22986	-	C1192_23975	IS66 family transposase
CDS	23064	23402	-	C1192_23980	IS66 family insertion sequence hypothetical
CDS	23396	23683	-	C1192_23985	IS66 family insertion sequence hypothetical
CDS	23787	24275	+	C1192_23990	IS630 family transposase
CDS	24308	24593	+	C1192_23995	IS110 family transposase
CDS	24592	24773	-	C1192_24000	IS110 family transposase
CDS	24895	25401	-	C1192_24005	IS256 family transposase
CDS	25474	26205	-	C1192_24010	phosphatase PAP2 family protein
CDS	26439	26629	+	C1192_24015	IS1 family transposase
CDS	26664	27182	-	C1192_24020	hypothetical protein
CDS	27342	27734	+	C1192_24025	DUF4158 domain-containing protein
CDS	27654	27875	-	C1192_24030	hypothetical protein
CDS	28409	29194	+	C1192_24035	IS630 family transposase
CDS	29529	30737	-	C1192_24040	IS256 family transposase
CDS	30847	30954	+	C1192_24045	hypothetical protein
CDS	31025	32845	-	C1192_24050	hypothetical protein
CDS	33328	33882	+	C1192_24055	ISNCY family transposase
CDS	34064	34291	+	C1192_24060	ISNCY family transposase
CDS	34478	36424	-	C1192_24065	DUF4116 domain-containing protein
CDS	37055	37306	+	C1192_24070	type II toxin-antitoxin system Phd/YefM family

Table S1 The gene lists of plasmid pEM148 in *E. marmotae* HT073016

CDS	37303	37557	+	C1192_24075	Txe/YoeB family addiction module toxin
CDS	37586	37843	-	C1192_24080	hypothetical protein
CDS	38373	38909	-	C1192_24085	general secretion pathway protein
CDS	38896	40089	-	C1192_24090	type II secretion system protein GspL
CDS	40086	41063	-	C1192_24095	type II secretion system protein
CDS	41063	41665	-	C1192_24100	type II secretion system protein GspJ
CDS	41662	42033	-	C1192_24105	type II secretion system protein GspI
CDS	42030	42593	-	C1192_24110	type II secretion system protein GspH
CDS	42597	43079	-	C1192_24115	type II secretion system protein GspG
CDS	43069	44292	-	C1192_24120	type II secretion system protein GspF
CDS	44292	45785	-	C1192_24125	type II secretion system protein GspE
CDS	45785	47845	-	C1192_24130	type II secretion system protein GspD
CDS	47875	48834	-	C1192_24135	type II secretion system protein GspC
CDS	48852	49262	-	C1192_24140	hypothetical protein
CDS	49328	50137	-	C1192_24145	prepilin peptidase
CDS	50329	54912	-	C1192_24150	DUF4092 domain-containing protein
CDS	55197	55904	-	C1192_24155	hypothetical protein
CDS	56692	58647	-	C1192_24160	hypothetical protein
CDS	59742	59984	-	C1192_24165	hypothetical protein
CDS	59997	61025	-	C1192_24170	EscU/YscU/HrcU family type III secretion system
CDS	61033	61800	-	C1192_24175	EscT/YscT/HrcT family type III secretion system
CDS	61800	62060	-	C1192_24180	EscS/YscS/HrcS family type III secretion system
CDS	62074	62724	-	C1192_24185	EscR/YscR/HrcR family type III secretion system
CDS	62714	63046	-	C1192_24190	hypothetical protein
CDS	63610	64518	-	C1192_24195	hypothetical protein
CDS	64499	64894	-	C1192_24200	hypothetical protein
CDS	64939	66231	-	C1192_24205	FliI/YscN family ATPase
CDS	66235	66636	-	C1192_24210	hypothetical protein
CDS	66648	68708	-	C1192_24215	EscV/YscV/HrcV family type III secretion system
CDS	68721	69794	-	C1192_24220	YopN family type III secretion system gatekeeper
CDS	69814	71511	-	C1192_24225	EscC/YscC/HrcC family type III secretion system
CDS	71498	72130	-	C1192_24230	transcriptional regulator
CDS	72257	72679	-	C1192_24235	hypothetical protein
CDS	72663	73070	-	C1192_24240	hypothetical protein
CDS	73045	73743	-	C1192_24245	MxiN
CDS	73709	74230	-	C1192_24250	protein mxiK
CDS	74235	74951	-	C1192_24255	EscJ/YscJ/HrcJ family type III secretion inner
CDS	74957	75250	-	C1192_24260	protein mxiI
CDS	75264	75515	-	C1192_24265	EscF/YscF/HrpA family type III secretion system
CDS	75526	76638	-	C1192_24270	protein mxiG
CDS	76655	77104	-	C1192_24275	invasion protein IagB
CDS	77101	77466	-	C1192_24280	molecular chaperone
CDS	77476	79089	-	C1192_24285	type III secretion system effector inositol
CDS	79399	80877	+	C1192_24290	virulence factor

Table S1 The gene lists of plasmid pEM148 in *E. marmotae* HT073016

CDS	80897	81286	+	C1192_24295	molecular chaperone
CDS	81303	81929	+	C1192_24300	protein ipgB
CDS	82000	82467	+	C1192_24305	type III secretion system translocator chaperone
CDS	82473	84209	+	C1192_24310	invasin
CDS	84224	85312	+	C1192_24315	IpaC/SipC family type III secretion system
CDS	85362	86357	+	C1192_24320	IpaD/SipD/SspD family type III secretion system
CDS	86366	90691	+	C1192_24325	hypothetical protein
CDS	90694	90918	+	C1192_24330	acyl carrier protein
CDS	91123	92049	+	C1192_24335	chromosome partitioning protein ParB
CDS	92225	92454	-	C1192_24340	hypothetical protein
CDS	92756	94327	-	C1192_24345	IS66 family transposase
CDS	94347	94694	-	C1192_24350	IS66 family insertion sequence hypothetical
CDS	94694	95349	-	C1192_24355	IS66 family insertion sequence hypothetical
CDS	95311	95421	+	C1192_24360	copper resistance protein
CDS	95640	96460	+	C1192_24365	IS3 family transposase
CDS	96582	97448	-	C1192_24370	invasion protein
CDS	97460	98200	-	C1192_24375	hypothetical protein
CDS	99150	99332	+	C1192_24380	AraC family transcriptional regulator
CDS	99446	99805	+	C1192_24385	hypothetical protein
tRNA	100074	100149	+	C1192_24390	tRNA-Met
tRNA	100191	100264	+	C1192_24395	tRNA-Arg
CDS	100541	104637	-	C1192_24400	autotransporter outer membrane beta-barrel
CDS	105080	105943	-	C1192_24405	Effector protein YopJ
CDS	106163	106282	-	C1192_24410	hypothetical protein
CDS	106497	107191	-	C1192_24415	IS1 family transposase
CDS	108221	117880	+	C1192_24420	DUF3491 domain-containing protein
CDS	118214	118318	-	C1192_24425	copper resistance protein
CDS	118280	118957	+	C1192_24430	IS66 family insertion sequence hypothetical
CDS	118957	119304	+	C1192_24435	IS66 family insertion sequence hypothetical
CDS	119324	120895	+	C1192_24440	IS66 family transposase
CDS	121533	121634	+	C1192_24445	pcar domain protein
CDS	121666	122290	-	C1192_24450	AAA family ATPase
CDS	122312	123637	-	C1192_24455	IS21 family transposase
CDS	123757	124224	-	C1192_24460	hypothetical protein
CDS	124231	125172	-	C1192_24465	shiE
CDS	125277	126427	+	C1192_24470	IS3 family transposase
CDS	126529	127230	+	C1192_24475	cell division protein
CDS	127884	128720	-	C1192_24480	IS3 family transposase
CDS	128794	130002	+	C1192_24485	IS256 family transposase
CDS	130041	130357	-	C1192_24490	IS3 family transposase
CDS	130412	130519	+	C1192_24495	IS66 family insertion sequence hypothetical
CDS	130601	131929	+	C1192_24500	IS4 family transposase
CDS	131943	132320	+	C1192_24505	IS66 family insertion sequence hypothetical
CDS	132320	132667	+	C1192_24510	IS66 family insertion sequence hypothetical

Table S1 The gene lists of plasmid pEM148 in *E. marmotae* HT073016

CDS	132687	134258	+	C1192_24515	IS66 family transposase
CDS	134284	134672	-	C1192_24520	IS1 family transposase
CDS	134929	135921	-	C1192_24525	nuclease PIN
CDS	135943	136458	-	C1192_24530	fimbrial protein
CDS	136533	137090	-	C1192_24535	nuclease PIN
CDS	137126	137857	-	C1192_24540	molecular chaperone
CDS	137926	140457	-	C1192_24545	PapC/FimD family outer membrane usher protein
CDS	140621	141208	-	C1192_24550	type 1 fimbrial protein
CDS	141288	141821	-	C1192_24555	type 1 fimbrial protein
CDS	142268	142573	-	C1192_24560	IS630 family transposase
CDS	142729	142950	-	C1192_24565	hypothetical protein
CDS	142980	144076	-	C1192_24570	conjugative transfer relaxase/helicase TraI
CDS	144227	144557	-	C1192_24575	conjugal transfer protein TraN
CDS	144636	144887	+	C1192_24580	type II toxin-antitoxin system Phd/YefM family
CDS	144884	145174	+	C1192_24585	type II toxin-antitoxin system RelE/ParE family
CDS	145246	145922	+	C1192_24590	IS1 family transposase
CDS	145971	146948	-	C1192_24595	chromosome partitioning protein ParB
CDS	146945	148150	-	C1192_24600	ParA family protein

Table S2 The gene lists of plasmid pEM76 in *E. marmotae* HT073016

	start	stop	strand	locus-tag	product
CDS	1	978	+	C1192_24605	repFIB replication protein A
CDS	1251	1991	-	C1192_24610	recombinase
CDS	2112	2240	-	C1192_24615	transcriptional regulator
CDS	2867	3667	+	C1192_24620	hypothetical protein
CDS	4220	4444	+	C1192_24625	type II toxin-antitoxin system Phd/YefM family
CDS	4441	4830	+	C1192_24630	type II toxin-antitoxin system death-on-curing
CDS	4922	5029	+	C1192_24635	integrase
CDS	4998	6170	+	C1192_24640	IS3 family transposase
CDS	6348	8645	+	C1192_24645	autotransporter outer membrane beta-barrel
CDS	8711	9919	-	C1192_24650	IS256 family transposase
CDS	10197	11984	-	C1192_24655	hypothetical protein
CDS	12598	12690	+	C1192_24660	LuxR family transcriptional regulator
CDS	12967	14655	-	C1192_24665	hypothetical protein
CDS	14933	15106	-	C1192_24670	IS630 family transposase
CDS	15441	16295	+	C1192_24675	hypothetical protein
CDS	16327	17741	-	C1192_24680	IS21 family transposase
CDS	17948	18199	-	C1192_24685	plasmid stabilization protein
CDS	18196	18395	-	C1192_24690	hypothetical protein
CDS	18553	18890	-	C1192_24695	hypothetical protein
CDS	18890	19081	+	C1192_24700	hypothetical protein
CDS	19053	19665	-	C1192_24705	type III secretion system protein
CDS	19874	20878	-	C1192_24710	integrase
CDS	20958	21347	-	C1192_24715	hypothetical protein
CDS	21324	22001	+	C1192_24720	IS66 family insertion sequence hypothetical
CDS	22001	22348	+	C1192_24725	IS66 family insertion sequence hypothetical
CDS	22368	23939	+	C1192_24730	IS66 family transposase
CDS	24797	25081	+	C1192_24735	hypothetical protein
CDS	25069	25554	+	C1192_24740	N-acetyltransferase
CDS	25682	26974	-	C1192_24745	IS4 family transposase
CDS	27059	27187	+	C1192_24750	IS66 family insertion sequence hypothetical
CDS	27213	28784	-	C1192_24755	IS66 family transposase
CDS	28804	29151	-	C1192_24760	IS66 family insertion sequence hypothetical
CDS	29151	29828	-	C1192_24765	IS66 family insertion sequence hypothetical
CDS	29949	30632	+	C1192_24770	IS66 family transposase
CDS	30636	30926	-	C1192_24775	type III effector
CDS	31197	31574	+	C1192_24780	hypothetical protein
CDS	32034	32675	+	C1192_24785	thiol:disulfide interchange protein
CDS	32689	33240	+	C1192_24790	hypothetical protein
CDS	33273	33539	-	C1192_24795	hypothetical protein
CDS	33719	33996	+	C1192_24800	IS3 family transposase
CDS	34835	39772	+	C1192_24805	inverse autotransporter beta-barrel
CDS	39890	40056	-	C1192_24810	IS256 family transposase
CDS	40215	40355	+	C1192_24815	IS630 family transposase

Table S2 The gene lists of plasmid pEM76 in *E. marmotae* HT073016

CDS	40547	41503	+	C1192_24820	IS481 family transposase
CDS	41645	42853	-	C1192_24825	IS256 family transposase
CDS	43166	44494	-	C1192_24830	IS4 family transposase
CDS	44551	46209	-	C1192_24835	E3 ubiquitin--protein ligase
CDS	46619	46699	-	C1192_24840	ferredoxin
CDS	46925	47017	-	C1192_24845	copper resistance protein
CDS	46994	47671	+	C1192_24850	IS66 family insertion sequence hypothetical
CDS	47671	48018	+	C1192_24855	IS66 family insertion sequence hypothetical
CDS	48038	49609	+	C1192_24860	IS66 family transposase
CDS	49641	50144	+	C1192_24865	IS3-like element ISEc16 family transposase
CDS	50827	50934	+	C1192_24870	hypothetical protein
CDS	51120	52924	-	C1192_24875	hypothetical protein
CDS	53344	54494	-	C1192_24880	IS3 family transposase
CDS	54566	54793	+	C1192_24885	IS630 family transposase
CDS	55064	55894	+	C1192_24890	AraC family transcriptional regulator
CDS	56157	56421	+	C1192_24895	IS630 family transposase
CDS	56533	56730	+	C1192_24900	IS256 family transposase
CDS	56889	57668	-	C1192_24905	AraC family transcriptional regulator
CDS	58478	59922	-	C1192_24910	hypothetical protein
CDS	60145	60924	-	C1192_24915	hypothetical protein
CDS	61526	62212	+	C1192_24920	virulence protein SpvD
CDS	62646	63365	+	C1192_24925	type III effector phosphothreonine lyase
CDS	64017	65167	+	C1192_24930	IS3 family transposase
CDS	65253	65852	-	C1192_24935	hypothetical protein
CDS	65898	66332	-	C1192_24940	DUF1380 domain-containing protein
CDS	66345	66566	-	C1192_24945	hypothetical protein
CDS	66567	67249	-	C1192_24950	DNA methylase
CDS	67575	68086	-	C1192_24955	hypothetical protein
CDS	68123	68391	-	C1192_24960	hypothetical protein
CDS	68776	69747	-	C1192_24965	plasmid-partitioning protein
CDS	69747	70913	-	C1192_24970	protein SopA
CDS	71501	72256	-	C1192_24975	replication initiation protein
CDS	73043	73831	-	C1192_24980	phage integrase family protein
CDS	73832	74137	-	C1192_24985	plasmid maintenance protein CcdB
CDS	74139	74357	-	C1192_24990	antitoxin CcdA
CDS	74359	74594	+	C1192_24995	hypothetical protein
CDS	74753	75338	+	C1192_25000	ISL3 family transposase
CDS	75621	75848	-	C1192_25005	hypothetical protein

Table S3 gene list of T3SS compared between *E.marmotae* and *Shigella*

Genes	Location	Query (locus)	Size(bp)	Pathogen hit	Location	Sbjct (locus)	Nucleotide identity(%)	Query cover(%)	Annotation (mechanism)
virB	pEM148	C1192_24335	927	*Shigella flexneri*	Plasmid pCP301 (NC_004851.1)	CP0123	80.13	99	transcriptional activator VirB
Acp	pEM148	C1192_24330	225	*Shigella flexneri*	Plasmid pCP301 (NC_004851.2)	CP0124	73.97	98	Acp, putative acyl carrier protein
ipaA (truncated)	pEM148	C1192_24325	4326	*Shigella flexneri*	Plasmid pCP301 (NC_004851.1)	CP0125	57.92	81	ipaA hypothetical protein
ipaD	pEM148	C1192_24320	996	*Shigella flexneri*	Plasmid pCP301 (NC_004851.1)	CP0126	77.13	98	ipaD hypothetical protein
ipaC	pEM148	C1192_24315	1089	*Shigella flexneri*	Plasmid pCP301 (NC_004851.1)	CP0127	70	82	IpaC, secreted by the Mxi-Spa secretion
ipaB	pEM148	C1192_24310	1737	*Shigella flexneri*	Plasmid pCP301 (NC_004851.1)	CP0128	70	93	IpaB, secreted by the Mxi-Spa secretion
ipgC	pEM148	C1192_24305	468	*Shigella flexneri*	Plasmid pCP301 (NC_004851.1)	CP0129	71	95	IpgC, cytoplasmic chaperone for IpaB and IpaC
ipgB1	pEM148	C1192_24300	627	*Shigella flexneri*	Plasmid pCP301 (NC_004851.1)	CP0130	79	100	protein IpgB1
ipgA	pEM148	C1192_24295	390	*Shigella flexneri*	Plasmid pCP301 (NC_004851.1)	CP0131	63.27	100	chaperone IpgA
icsB	pEM148	C1192_24290	1479	*Shigella flexneri*	Plasmid pCP301 (NC_004851.1)	CP0132	65	77	IcsB, invasion protein
ipgD	pEM148	C1192_24285	1614	*Shigella flexneri*	Plasmid pCP301 (NC_004851.1)	CP0133	68	64	hypothetical protein
ipgE	pEM148	C1192_24280	366	*Shigella flexneri*	Plasmid pCP301 (NC_004851.1)	CP0134	69	77	IpgE, cytoplasmic chaperone for IpgD
ipgF	pEM148	C1192_24275	450	*Shigella flexneri*	Plasmid pCP301 (NC_004851.1)	CP0135	72	95	periplasmic protein IpgF
mxiG	pEM148	C1192_24270	1113	*Shigella flexneri*	Plasmid pCP301 (NC_004851.1)	CP0136	78	85	MxiG protein
mxiH	pEM148	C1192_24265	252	*Shigella flexneri*	Plasmid pCP301 (NC_004851.1)	CP0137	71	74	Mxi-Spa secretion machinery protein MxiH
mxiI	pEM148	C1192_24260	294	*Shigella flexneri*	Plasmid pCP301 (NC_004851.1)	CP0138	76	100	Mxi-Spa secretion machinery protein MxiI
mxiJ	pEM148	C1192_24255	717	*Shigella flexneri*	Plasmid pCP301 (NC_004851.1)	CP0139	67	97	Mxi-Spa secretion machinery protein MxiJ
mxiK	pEM148	C1192_24250	522	*Shigella flexneri*	Plasmid pCP301 (NC_004851.1)	CP0140	78	89	Mxi-Spa secretion machinery protein MxiK
mxiN	pEM148	C1192_24245	699	*Shigella flexneri*	Plasmid pCP301 (NC_004851.1)	CP0141	71	78	Mxi-Spa secretion machinery protein MxiN
mxiL	pEM148	C1192_24240	408	*Shigella flexneri*	Plasmid pCP301 (NC_004851.1)	CP0142	74	71	Mxi-Spa secretion machinery protein MxiL
mxiM	pEM148	C1192_24235	423	*Shigella flexneri*	Plasmid pCP301 (NC_004851.1)	CP0143	69	49	Mxi-Spa secretion machinery protein MxiM
mxiE	pEM148	C1192_24230	633	*Shigella flexneri*	Plasmid pCP301 (NC_004851.1)	CP0144	70	98	AraC-like family protein MxiE
mxiD	pEM148	C1192_24225	1698	*Shigella flexneri*	Plasmid pCP301 (NC_004851.1)	CP0145	75	90	outermembrane protein MxiD
mxiC	pEM148	C1192_24220	1074	*Shigella flexneri*	Plasmid pCP301 (NC_004851.1)	CP0146	72.38	99	Mxi-Spa secretion machinery protein
mxiA	pEM148	C1192_24215	2061	*Shigella flexneri*	Plasmid pCP301 (NC_004851.1)	CP0147	76.52	99	Mxi-Spa secretion machinery protein MxiA
spa15	pEM148	C1192_24210	402	*Shigella flexneri*	Plasmid pCP301 (NC_004851.1)	CP0148	75.87	97	Mxi-Spa secretion machinery protein Spa15
spa47	pEM148	C1192_24205	1293	*Shigella flexneri*	Plasmid pCP301 (NC_004851.1)	CP0149	75	97	ATP synthase SpaL
spa13	pEM149	C1192_24200	396	*Shigella flexneri*	Plasmid pCP301 (NC_004851.1)	CP0150	44.64	85	Mxi-Spa secretion machinery protein Spa13
spa32	pEM148	C1192_24195	909	*Shigella flexneri*	Plasmid pCP301 (NC_004851.1)	CP0151	71	69	Mxi-Spa secretion machinery protein Spa32
spa33	pEM148	C1192_24190	333	*Shigella flexneri*	Plasmid pCP301 (NC_004851.1)	CP0152	67	49	Mxi-Spa secretion machinery protein Spa33
spa24	pEM148	C1192_24185	651	*Shigella flexneri*	Plasmid pCP301 (NC_004851.1)	CP0153	78	97	surface presentation of antigens protein SpaP
spa9	pEM148	C1192_24180	261	*Shigella flexneri*	Plasmid pCP301 (NC_004851.2)	CP0154	94.12	98	Mxi-Spa secretion machinery protein Spa9
spa29	pEM148	C1192_24175	768	*Shigella flexneri*	Plasmid pCP301 (NC_004851.1)	CP0155	72	98	Mxi-Spa secretion machinery protein Spa29
spa40	pEM148	C1192_24170	1029	*Shigella flexneri*	Plasmid pCP301 (NC_004851.1)	CP0156	74.15	100	surface presentation of antigens protein SpaS
OspD2	pEM148	missing	\	*Shigella flexneri*	Plasmid pCP301 (NC_004851.1)	CP0009	\	\	OspD2 hypothetical protein
OspF	pEM148	missing	\	*Shigella flexneri*	Plasmid pCP301 (NC_004851.1)	CP0010	\	\	OspF
OspD1	pEM148	missing	\	*Shigella flexneri*	Plasmid pCP301 (NC_004851.1)	CP0022	\	\	protein OspD1
ipgB2	pEM148	missing	\	*Shigella flexneri*	Plasmid pCP301 (NC_004851.1)	CP0024	\	\	ipgB2 hypothetical protein
VirF	pEM148	missing	\	*Shigella flexneri*	Plasmid pCP301 (NC_004851.1)	CP0046	\	\	transcriptional activator VirF
OspE2	pEM148	missing	\	*Shigella flexneri*	Plasmid pCP301 (NC_004851.1)	CP0048	\	\	OspE2
OspD3	pEM148	missing	\	*Shigella flexneri*	Plasmid pCP301 (NC_004851.1)	CP0093	\	\	OspD3 hypothetical protein
OspC1	pEM148	missing	\	*Shigella flexneri*	Plasmid pCP301 (NC_004851.1)	CP0094	\	\	protein OspC1
OspC3	pEM148	missing	\	*Shigella flexneri*	Plasmid pCP301 (NC_004851.1)	CP0115	\	\	OspC3

Table S3 gene list of T3SS compared between *E.marmotae* and *Shigella*

icsA/virG	pEM148	missing	\	*Shigella flexneri*	Plasmid pCP301 (NC_004851.1)	CP0182	\	\	icsA/virG hypothetical protein
OspG	pEM148	missing	\	*Shigella flexneri*	Plasmid pCP301 (NC_004851.1)	CP0227	\	\	protein OspG
OspE1	pEM148	missing	\	*Shigella flexneri*	Plasmid pCP301 (NC_004851.1)	CP0265a	\	\	OspE1

Table S4The virulent genes of *E. marmotae* HT073016

	Genes	Location	Size(bp)	Pathogen hit	Location	Nucleotide identity(%)	Query cover(%)	Annotation (mechanism)	Reference
O antigen gene cluster	*galF*	chromosome	894	*Escherichia coli*	Chromosome (AB811615.1)	99	100	UTP-glucose-1-phosphate uridylyltransferase	1
	wffK	chromosome	816	*Escherichia coli*	Chromosome (AB811615.1)	100	100	Glycosyl transferase	1
	wzy	chromosome	1227	*Escherichia coli*	Chromosome (AB811615.1)	99	100	O-antigen polymerase	1
	wzx	chromosome	1197	*Escherichia coli*	Chromosome (AB811615.1)	99	100	O-antigen flippase	1
	wffL	chromosome	867	*Escherichia coli*	Chromosome (AB811615.1)	99	100	Glycosyl transferase	1
	wffM	chromosome	687	*Escherichia coli*	Chromosome (AB811615.1)	100	100	Glycosyltransferase sugar-binding region	1
	galE	chromosome	1032	*Escherichia coli*	Chromosome (AB811615.1)	99	100	UDP-glucose 4-epimerase	1
	wfgD	chromosome	747	*Escherichia coli*	Chromosome (AB811615.1)	99	100	UDP-Glc:alpha-D-GlcNAc-diphosphoundecaprenol beta-1,3-glucosyltransferase	1
Type II secretion system	*gspM*	pEM148	537	*Escherichia coli*	Chromosome (CP018976.1)	85	100	Inner-membrane platform protein	2
	gspL	pEM148	1194	*Escherichia coli*	Chromosome (CP018976.1)	87	98	Inner-membrane platform protein	2
	gspK	pEM148	981	*Escherichia coli*	Chromosome (CP018976.1)	91	99	Minor pseudopilin	2
	gspJ	pEM148	567	*Escherichia coli*	Chromosome (CP018976.1)	89	99	Minor pseudopilin	2
	gspI	pEM148	372	*Escherichia coli*	Chromosome (CP018976.1)	89	100	Minor pseudopilin	2
	gspH	pEM148	564	*Escherichia coli*	Chromosome (CP018976.1)	94	100	Minor pseudopilin	2
	gspG	pEM148	456	*Escherichia coli*	Chromosome (CP018976.1)	91	100	Minor pseudopilin	2
	gspF	pEM148	1224	*Escherichia coli*	Chromosome (CP018976.1)	93	100	Inner-membrane platform protein	2
	gspE	pEM148	1494	*Escherichia coli*	Chromosome (CP018976.1)	95	100	Secretion ATPase	2
	gspD	pEM148	2061	*Escherichia coli*	Chromosome (CP018976.1)	94	100	Outer-membrane secretin	2
	gspC	pEM148	960	*Escherichia coli*	Chromosome (CP018976.1)	90	100	Inner-membrane platform protein	2
sfp cluster	*sfpA*	pEM148	534	*Escherichia coli*	Plasmid pCFSAN004177_01 (CP012496.1)	85	100	Fimbrial protein precursor SfpA	3
	sfpH	pEM148	588	*Escherichia coli*	Plasmid pCFSAN004177_01 (CP012496.1)	90	100	Fimbrial protein SfpH	3
	sfpC	pEM148	2532	*Escherichia coli*	Plasmid pCFSAN004177_01 (CP012496.1)	92	100	Chaperone	3
	sfpD	pEM148	732	*Escherichia coli*	Plasmid pCFSAN004177_01 (CP012496.1)	93	100	Fimbrial protein SfpD	3
	sfpJ	pEM148	558	*Escherichia coli*	Plasmid pCFSAN004177_01 (CP012496.1)	82	100	Fimbrial protein SfpJ	3
	sfpF	pEM148	516	*Escherichia coli*	Plasmid pCFSAN004177_01 (CP012496.1)	86	100	Fimbrial protein SfpF	3
	sfpG	pEM148	993	*Escherichia coli*	Plasmid pCFSAN004177_01 (CP012496.1)	86	100	Fimbrial protein SfpG	3

Table S4The virulent genes of *E. marmotae* HT073016

ipaH	*ipaH1*	pEM148	1683	*Shigella flexneri*	Chromosome (CP004057)	74	54		4
	ipaH2	pEM148	2064	*Shigella flexneri*	Chromosome (CP020086)	75	45		4
	ipaH3	pEM148	2292	-	-	-	-		4
	ipaH4	pEM148	1619	-	-	-	-		4
	ipaH5	pEM76	801	-	-	-	-		4
	ipaH6	pEM76	2274	*Shigella sonnei*		84	43		4
	ipaH7	pEM76	1689	-	-	-	-		4
	ipaH8	pEM76	1659	*Shigella flexneri*	Plasmid pCP301 (NC_004851.1)	92	99		4
	ipaH9	pEM76	1977	*Shigella dysenteriae*		74	44		4
	ipaH10	chromosome	2304	*Shigella flexneri*		93	40		4
	ipaH11	chromosome	1824	*Shigella flexneri*		92	51		4
	yopJ	pEM148	864	*Yersinia enterocolitica*	Chromosome（NP_395205）	82	98	Effector protein yopJ	5
	lifA/efa	pEM148	9660	*Escherichia coli*	Chromosome (CP015241.1)	89	100	Adherence protein	6
	cif	pEM148	702	*Escherichia coli*	Chromosome (CP020107.1)	74	92	T3SS effector cycle inhibiting factor	7
	epeA	pEM148	3690	*Escherichia coli*	Plasmid Po113 (AY258503.2)	74	77	Autotransporter protease	8
	ospC4	pEM76	1262	*Shigella flexneri*	Plasmid pCP301 (NC_004851.1)	79	98	OspC4	9
	astA	chromosome	117	*Escherichia coli*	PlasmidAM1 (AF143819)	100	100		10
		pEM148	117	*Escherichia coli*	PlasmidAM1(AF143819)	100	100		10
		Pem76	117	*Escherichia coli*	PlasmidAM1(AF143819)	100	100		10

reference

1 Iguchi, A., et al., A complete view of the genetic diversity of the *Escherichia coli* O-antigen biosynthesis gene cluster. DNA Res, 2015. 22: p. 101-7.

2 Korotkov, K.V., M. Sandkvist, and W.G. Hol, The type II secretion system: biogenesis, molecular architecture and mechanism. Nat Rev Microbiol, 2012. 10: p. 336-51.

3 Musken, A., et al., Anaerobic conditions promote expression of Sfp fimbriae and adherence of sorbitol-fermenting enterohemorrhagic *Escherichia coli* O157:NM to human intestinal epithelial cells. Appl Environ Microbiol, 2008. 74: p. 1087-93.

4 Li, P., et al., Ubiquitination and degradation of GBPs by a *Shigella* effector to suppress host defence. Nature, 2017. 551: p. 378-383.

5 Mukherjee, S., et al., *Yersinia* YopJ acetylates and inhibits kinase activation by blocking phosphorylation. Science, 2006. 312: p. 1211-4.

6 Badea, L., et al., Contribution of Efa1/LifA to the adherence of enteropathogenic *Escherichia coli* to epithelial cells. Microb Pathog, 2003. 34: p. 205-15.

7 Hsu, Y., et al., Structure of the cyclomodulin Cif from pathogenic *Escherichia coli*. J Mol Biol, 2008. 384: p. 465-77.

8 Leyton, D.L., et al., Transfer region of pO113 from enterohemorrhagic *Escherichia coli*: similarity with R64 and identification of a novel plasmid-encoded autotransporter, EpeA. Infect Immun, 2003. 71: p. 6307-19.

9 Jin, Q., et al., Genome sequence of *Shigella flexneri* 2a: insights into pathogenicity through comparison with genomes of *Escherichia coli* K12 and O157. Nucleic Acids Research, 2002. 30: p. 4432-4441.

10 A, M., et al., IS1414, an *Escherichia coli* insertion sequence with a heat-stable enterotoxin gene embedded in a transposase-like gene. . Infect Immun, 2000 68: p. 5710-5715.

Table S5 BLASTP of *E. marmotae* putative type VI secretion system (T6SS) locus variable regions.							
**T6SS1**							
**similarity between *E.marmotae* and *Salmonella***							
**strain**	**Query (locus)**	**% identity**	**E-value**	**strain**	**Sbjct (locus)**	**Accession**	**Description**
***E. marmotae* HT073016**	C1192_RS11705	80.25%	0	***Salmonella enterica* subsp. enterica serovar Typhimurium str. LT2**	STM0289	NP_459287.1	type VI secretion system tip protein VgrG
***E. marmotae* HT073016**	C1192_RS11710	N		***Salmonella enterica* subsp. enterica serovar Typhimurium str. LT2**		NP_459286.1	putative cytoplasmic protein
***E. marmotae* HT073016**	C1192_RS11715	N		***Salmonella enterica* subsp. enterica serovar Typhimurium str. LT2**		NP_459285.1	putative cytoplasmic protein
***E. marmotae* HT073016**	C1192_RS11720	74.80%	7.00E-137	***Salmonella enterica* subsp. enterica serovar Typhimurium str. LT2**	STM0286	NP_459284.1	hypothetical protein
***E. marmotae* HT073016**	C1192_RS11725	86.31%	0	***Salmonella enterica* subsp. enterica serovar Typhimurium str. LT2**	STM0285	NP_459283.1	type VI secretion system membrane subunit TssM
***E. marmotae* HT073016**	C1192_RS11730	N		***Salmonella enterica* subsp. enterica serovar Typhimurium str. LT2**		NP_459282.1	hypothetical protein
***E. marmotae* HT073016**	C1192_RS11735	50.00%	0.058	***Salmonella enterica* subsp. enterica serovar Typhimurium str. LT2**	STM0283	NP_459281.1	hypothetical protein
***E. marmotae* HT073016**	C1192_RS11740	79.00%	0	***Salmonella enterica* subsp. enterica serovar Typhimurium str. LT2**	STM0282	NP_459280.1	type VI secretion system protein TssL
***E. marmotae* HT073016**	C1192_RS11745	85.23%	0	***Salmonella enterica* subsp. enterica serovar Typhimurium str. LT2**	STM0281	NP_459279.1	type VI secretion system baseplate subunit TssK
***E. marmotae* HT073016**	C1192_RS11750	74.30%	1.00E-94	***Salmonella enterica* subsp. enterica serovar Typhimurium str. LT2**	STM0280	NP_459278.1	type VI secretion system lipoprotein
***E. marmotae* HT073016**		N		***Salmonella enterica* subsp. enterica serovar Typhimurium str. LT2**	STM0279	NP_459277.1	Hcp1 family type VI secretion system effector
***E. marmotae* HT073016**		N		***Salmonella enterica* subsp. enterica serovar Typhimurium str. LT2**	STM0278	NP_459276.1	putative periplasmic protein
***E. marmotae* HT073016**		N		***Salmonella enterica* subsp. enterica serovar Typhimurium str. LT2**	STM0277	NP_459275.1	hypothetical protein
***E. marmotae* HT073016**	C1192_RS11755	95.03%	6.00E-120	***Salmonella enterica* subsp. enterica serovar Typhimurium str. LT2**	STM0276	NP_459274.1	type VI secretion system tube protein Hcp
***E. marmotae* HT073016**	C1192_RS11760	50.91%	1.00E-43	***Salmonella enterica* subsp. enterica serovar Typhimurium str. LT2**	STM0275	NP_459273.3	putative cytoplasmic protein
***E. marmotae* HT073016**	C1192_RS11765	68.75%	1.00E-14	***Salmonella enterica* subsp. enterica serovar Typhimurium str. LT2**	STM0274a	NP_459272.1	invasol SirA
***E. marmotae* HT073016**	C1192_RS11770	93.06%	0	***Salmonella enterica* subsp. enterica serovar Typhimurium str. LT2**	STM0274	NP_459271.1	type VI secretion system contractile sheath large subunit
***E. marmotae* HT073016**	C1192_RS11775	93.60%	6.00E-121	***Salmonella enterica* subsp. enterica serovar Typhimurium str. LT2**	STM0273	NP_459270.1	type VI secretion system contractile sheath small subunit
***E. marmotae* HT073016**	C1192_RS11780	84.28%	0	***Salmonella enterica* subsp. enterica serovar Typhimurium str. LT2**	STM0272	NP_459269.1	type VI secretion system ATPase

Table S5 BLASTP of *E. marmotae* putative type VI secretion system (T6SS) locus variable regions.							
***E. marmotae* HT073016**	C1192_RS11785	57.57%	6.00E-124	***Salmonella enterica* subsp. enterica serovar Typhimurium str. LT2**	STM0271	NP_459268.1	type VI secretion system-associated protein
***E. marmotae* HT073016**	C1192_RS11790	71.01%	4.00E-151	***Salmonella enterica* subsp. enterica serovar Typhimurium str. LT2**	STM0270	NP_459267.1	impE family protein
***E. marmotae* HT073016**	C1192_RS11795	76.22%	5.00E-95	***Salmonella enterica* subsp. enterica serovar Typhimurium str. LT2**	STM0269	NP_459266.1	type VI secretion system baseplate subunit TssE
***E. marmotae* HT073016**	C1192_RS11800	89.63%	0.00E+00	***Salmonella enterica* subsp. enterica serovar Typhimurium str. LT2**	STM0268	NP_459265.1	type VI secretion system baseplate subunit TssF
***E. marmotae* HT073016**	C1192_RS11805	83.99%	0.00E+00	***Salmonella enterica* subsp. enterica serovar Typhimurium str. LT2**	STM0267	NP_459264.1	type VI secretion system baseplate subunit TssG
***E. marmotae* HT073016**	C1192_RS11810	72.44%	2.00E-173	***Salmonella enterica* subsp. enterica serovar Typhimurium str. LT2**	STM0266	NP_459263.1	hypothetical protein
**T6SS2**							
**similarity between *E.marmotae* and *E.coli***							
**strain**	**Query (locus)**	**% identity**	**E-value**	**strain**	**Sbjct (locus)**	**Accession**	**Description**
***E. marmotae* HT073016**	C1192_RS20665	73.17%	5.00E-87	***Escherichia coli* 042**	EC042_RS24170	WP_000098939.1	type VI secretion system contractile sheath small subunit
***E. marmotae* HT073016**	C1192_RS20670	85.83%	0.00E+00	***Escherichia coli* 042**	EC042_RS24175	WP_000930191.1	type VI secretion system contractile sheath large subunit
***E. marmotae* HT073016**	C1192_RS20675	73.65%	0.00E+00	***Escherichia coli* 042**	EC042_RS24180	WP_000708638.1	type VI secretion system baseplate subunit TssK
***E. marmotae* HT073016**	C1192_RS20680	63.21%	3.00E-99	***Escherichia coli* 042**	EC042_RS24185	WP_001040045.1	type VI secretion system protein ImpK
***E. marmotae* HT073016**	C1192_RS20685	48.18%	1.00E-178	***Escherichia coli* 042**	EC042_RS24190	WP_000558483.1	cell envelope biogenesis protein OmpA
***E. marmotae* HT073016**	C1192_RS20690	94.48%	6.00E-112	***Escherichia coli* 042**	EC042_RS24195	WP_001007313.1	Hcp family type VI secretion system effector
***E. marmotae* HT073016**	C1192_RS20695	79.55%	0.00%	***Escherichia coli* 042**	EC042_RS24200	WP_000431646.1	type VI secretion system ATPase TssH
***E. marmotae* HT073016**	C1192_RS20700	29.17%	0.21	***Escherichia coli* 042**	EC042_RS24205	WP_000148361.1	hypothetical protein
***E. marmotae* HT073016**	N	N		***Escherichia coli* 042**	EC042_RS28850	WP_001403984.1	hypothetical protein
***E. marmotae* HT073016**	N	N		***Escherichia coli* 042**	EC042_RS24215	WP_001054503.1	type VI secretion system tip protein VgrG
***E. marmotae* HT073016**	N	N		***Escherichia coli* 042**	EC042_RS24220	WP_000170556.1	DUF2235 domain-containing protein
***E. marmotae* HT073016**	N	N		***Escherichia coli* 042**	EC042_RS24225	WP_000196359.1	DUF2931 family protein
***E. marmotae* HT073016**	N	N		***Escherichia coli* 042**	EC042_RS24230	WP_000196358.1	DUF2931 family protein
***E. marmotae* HT073016**	N	N		***Escherichia coli* 042**	EC042_RS24235	WP_000033410.1	PAAR domain-containing protein

Table S5 BLASTP of *E. marmotae* putative type VI secretion system (T6SS) locus variable regions.							
***E. marmotae* HT073016**	N	N		***Escherichia coli* 042**	EC042_RS24240	WP_001403985.1	hypothetical protein
***E. marmotae* HT073016**	N	N		***Escherichia coli* 042**	EC042_RS24245	WP_001037088.1	type VI secretion protein VasK
***E. marmotae* HT073016**	N	N		***Escherichia coli* 042**	EC042_RS24250	WP_000146515.1	type VI secretion system baseplate subunit TssA
***E. marmotae* HT073016**	N	N		***Escherichia coli* 042**	EC042_RS24255	WP_000115357.1	hypothetical protein
***E. marmotae* HT073016**	N	N		***Escherichia coli* 042**	EC042_RS24260	WP_000342463.1	type VI secretion system baseplate subunit TssF
***E. marmotae* HT073016**	N	N		***Escherichia coli* 042**	EC042_RS24265	WP_000553781.1	type VI secretion system baseplate subunit TssG
***E. marmotae* HT073016**	N	N		***Escherichia coli* 042**	EC042_RS24270	WP_000974469.1	type VI secretion system lipoprotein TssJ
***E. marmotae* HT073016**	N	N		***Escherichia coli* 042**	EC042_RS24275	WP_001137932.1	type VI secretion system baseplate subunit TssE
***E. marmotae* HT073016**	N	N		***Escherichia coli* 042**	EC042_RS24360	WP_000072549.1	type VI secretion system contractile sheath small subunit
***E. marmotae* HT073016**	N	N		***Escherichia coli* 042**	EC042_RS24365	WP_000215053.1	type VI secretion system contractile sheath large subunit
***E. marmotae* HT073016**	N	N		***Escherichia coli* 042**	EC042_RS24370	WP_000233190.1	type VI secretion system protein AaiC/Hcp2
***E. marmotae* HT073016**	N	N		***Escherichia coli* 042**	EC042_RS24375	WP_000555548.1	hypothetical protein
***E. marmotae* HT073016**	N	N		***Escherichia coli* 042**	EC042_RS24380	WP_001390300.1	type VI secretion system baseplate subunit TssF
***E. marmotae* HT073016**	N	N		***Escherichia coli* 042**	EC042_RS24385	WP_001173973.1	type VI secretion system baseplate subunit TssG
***E. marmotae* HT073016**	N	N		***Escherichia coli* 042**	EC042_RS24390	WP_000198270.1	type VI secretion system tip protein VgrG
***E. marmotae* HT073016**	N	N		***Escherichia coli* 042**	EC042_RS24395	WP_000005080.1	hypothetical protein
***E. marmotae* HT073016**	N	N		***Escherichia coli* 042**	EC042_RS24400	WP_001390299.1	type VI secretion protein
***E. marmotae* HT073016**	N	N		***Escherichia coli* 042**	EC042_RS24405	WP_001033155.1	type VI secretion system protein TssA
***E. marmotae* HT073016**	N	N		***Escherichia coli* 042**	EC042_RS24410	WP_000152745.1	type VI secretion protein
***E. marmotae* HT073016**	N	N		***Escherichia coli* 042**	EC042_RS24415	WP_001154665.1	type VI secretion system baseplate subunit TssK
***E. marmotae* HT073016**	N	N		***Escherichia coli* 042**	EC042_RS24420	WP_000480661.1	hypothetical protein
***E. marmotae* HT073016**	N	N		***Escherichia coli* 042**	EC042_RS24425	WP_001028111.1	DotU family type IV/VI secretion system protein

Table S5 BLASTP of *E. marmotae* putative type VI secretion system (T6SS) locus variable regions.							
***E. marmotae* HT073016**	N	N		***Escherichia coli* 042**	EC042_RS24430	WP_001403210.1	hypothetical protein
***E. marmotae* HT073016**	N	N		***Escherichia coli* 042**	EC042_RS24435	WP_000029849.1	type VI secretion system ATPase TssH

Table S6 IS location in the genome of *E. marmotae* HT073016						
	**IS_name**	**IS family**	**locus tag**	**location**	**orf_L**	**orf_R**
1	IS4	IS4	0730-16_genome_1-orf83	chromosome	73291	71963
2		IS4	0730-16_genome_1-orf204	chromosome	215046	213718
3		IS4	0730-16_genome_1-orf419	chromosome	449338	448010
4		IS4	0730-16_genome_1-orf566	chromosome	569485	568157
5		IS4	0730-16_genome_1-orf830	chromosome	827342	826014
6		IS4	0730-16_genome_1-orf934	chromosome	941429	940101
7		IS4	0730-16_genome_1-orf939	chromosome	944730	946058
8		IS4	0730-16_genome_1-orf1097	chromosome	1108754	1108494
8		IS4	0730-16_genome_1-orf1098	chromosome	1109822	1108755
9		IS4	0730-16_genome_1-orf1155	chromosome	1171219	1169891
10		IS4	0730-16_genome_1-orf1183	chromosome	1195542	1196870
11		IS4	0730-16_genome_1-orf1303	chromosome	1306329	1307261
11		IS4	0730-16_genome_1-orf1304	chromosome	1307298	1307657
12		IS4	0730-16_genome_1-orf1314	chromosome	1315544	1314216
13		IS4	0730-16_genome_1-orf1415	chromosome	1424977	1426305
14		IS4	0730-16_genome_1-orf1543	chromosome	1586790	1585462
15		IS4	0730-16_genome_1-orf1760	chromosome	1804918	1806246
16		IS4	0730-16_genome_1-orf1864	chromosome	1920034	1918706
17		IS4	0730-16_genome_1-orf2623	chromosome	2722004	2720676
18		IS4	0730-16_genome_1-orf2904	chromosome	3001929	3000601
19		IS4	0730-16_genome_1-orf2946	chromosome	3055682	3055422
19		IS4	0730-16_genome_1-orf2947	chromosome	3056751	3055675
20		IS4	0730-16_genome_1-orf3368	chromosome	3528172	3529500
21		IS4	0730-16_genome_1-orf3487	chromosome	3672103	3673431
22		IS4	0730-16_genome_1-orf3492	chromosome	3678166	3676838
23		IS4	0730-16_genome_1-orf3659	chromosome	3853281	3854609
24		IS4	0730-16_genome_1-orf3734	chromosome	3933172	3932636
24		IS4	0730-16_genome_1-orf3735	chromosome	3933963	3933148
25		IS4	0730-16_genome_1-orf3742	chromosome	3942614	3943942
26		IS4	0730-16_genome_1-orf3746	chromosome	3947597	3946269
27		IS4	0730-16_genome_1-orf3831	chromosome	4034839	4036167
28		IS4	0730-16_genome_1-orf3895	chromosome	4094944	4096272
29		IS4	0730-16_genome_1-orf4047	chromosome	4271974	4273302
30		IS4	0730-16_genome_1-orf4142	chromosome	4366888	4368216
31		IS4	0730-16_genome_1-orf4190	chromosome	4412667	4411339
32		IS4	0730-16_genome_1-orf4345	chromosome	4591695	4593023
33		IS4	0730-16_genome_1-orf4349	chromosome	4596667	4595504
34		IS4	Contig5-orf9	pEM148	7698	9026
35		IS4	Contig6-orf41	pEM76	37421	38749
36		IS4	Contig6-orf55	pEM76	54941	56233
1	IS*1414*	IS256	0730-16_genome_1-orf201	chromosome	211298	210090
2		IS256	0730-16_genome_1-orf294	chromosome	323622	324830
3		IS256	0730-16_genome_1-orf806	chromosome	809570	810778

Table S6 IS location in the genome of *E. marmotae* HT073016						
4		IS256	0730-16_genome_1-orf866	chromosome	858892	857684
5		IS256	0730-16_genome_1-orf935	chromosome	941547	942755
6		IS256	0730-16_genome_1-orf1064	chromosome	1072022	1073230
7		IS256	0730-16_genome_1-orf1096	chromosome	1107227	1108435
8		IS256	0730-16_genome_1-orf1312	chromosome	1313989	1312781
9		IS256	0730-16_genome_1-orf1596	chromosome	1642595	1643803
10		IS256	0730-16_genome_1-orf1682	chromosome	1714252	1715460
11		IS256	0730-16_genome_1-orf1759	chromosome	1804801	1803593
12		IS256	0730-16_genome_1-orf1863	chromosome	1917405	1918613
13		IS256	0730-16_genome_1-orf1898	chromosome	1952080	1953288
14		IS256	0730-16_genome_1-orf1900	chromosome	1955621	1956829
15		IS256	0730-16_genome_1-orf2011	chromosome	2073721	2072513
16		IS256	0730-16_genome_1-orf2179	chromosome	2242374	2241166
17		IS256	0730-16_genome_1-orf2217	chromosome	2285689	2286897
18		IS256	0730-16_genome_1-orf2514	chromosome	2620328	2619120
19		IS256	0730-16_genome_1-orf2599	chromosome	2704232	2705440
20		IS256	0730-16_genome_1-orf3218	chromosome	3355946	3357154
21		IS256	0730-16_genome_1-orf3486	chromosome	3670766	3671974
22		IS256	0730-16_genome_1-orf3490	chromosome	3675863	3674655
23		IS256	0730-16_genome_1-orf3603	chromosome	3792649	3791441
24		IS256	0730-16_genome_1-orf3630	chromosome	3823084	3821876
25		IS256	0730-16_genome_1-orf3731	chromosome	3930216	3929008
26		IS256	0730-16_genome_1-orf3745	chromosome	3946172	3944964
27		IS256	0730-16_genome_1-orf3894	chromosome	4093607	4094815
28		IS256	0730-16_genome_1-orf4048	chromosome	4274838	4273630
29		IS256	0730-16_genome_1-orf4083	chromosome	4307967	4306759
30		IS256	0730-16_genome_1-orf4143	chromosome	4368313	4369521
31		IS256	0730-16_genome_1-orf4344	chromosome	4590358	4591566
32		IS256	0730-16_genome_1-orf4348	chromosome	4595455	4594247
33		IS256	Contig5-orf7	pEM148	5891	7099
34		IS256	Contig5-orf33	pEM148	29903	31111
35		IS256	Contig5-orf63	pEM148	55435	56643
36		IS256	Contig6-orf42	pEM76	39062	40270
37		IS256	Contig6-orf73	pEM76	71996	73204
1	ISCro1	IS66	0730-16_genome_1-orf507	chromosome	522497	520926
1		IS66	0730-16_genome_1-orf508	chromosome	522864	522517
1		IS66	0730-16_genome_1-orf509	chromosome	523511	522864
2		IS66	0730-16_genome_1-orf565	chromosome	567862	568146
2		IS66	0730-16_genome_1-orf567	chromosome	569642	569947
2		IS66	0730-16_genome_1-orf567	chromosome	569947	570294
2		IS66	0730-16_genome_1-orf569	chromosome	570314	571885
3		IS66	0730-16_genome_1-orf581	chromosome	583087	583734
3		IS66	0730-16_genome_1-orf582	chromosome	583734	584081
3		IS66	0730-16_genome_1-orf583	chromosome	584101	585672

Table S6 IS location in the genome of *E. marmotae* HT073016						
4		IS66	0730-16_genome_1-orf829	chromosome	825719	826003
4		IS66	0730-16_genome_1-orf831	chromosome	827499	827804
4		IS66	0730-16_genome_1-orf832	chromosome	827804	828151
4		IS66	0730-16_genome_1-orf833	chromosome	828171	829742
5		IS66	0730-16_genome_1-orf938	chromosome	944364	944648
5		IS66	0730-16_genome_1-orf940	chromosome	946144	946449
5		IS66	0730-16_genome_1-orf941	chromosome	946449	946796
5		IS66	0730-16_genome_1-orf942	chromosome	946816	948387
6		IS66	0730-16_genome_1-orf988	chromosome	989027	989512
6		IS66	0730-16_genome_1-orf989	chromosome	989512	989859
6		IS66	0730-16_genome_1-orf990	chromosome	989879	991450
7		IS66	0730-16_genome_1-orf1152	chromosome	1169133	1167562
7		IS66	0730-16_genome_1-orf1153	chromosome	1169500	1169153
7		IS66	0730-16_genome_1-orf1154	chromosome	1169805	1169500
8		IS66	0730-16_genome_1-orf1211	chromosome	1220567	1221052
8		IS66	0730-16_genome_1-orf1212	chromosome	1221052	1221399
8		IS66	0730-16_genome_1-orf1213	chromosome	1221419	1222990
9		IS66	0730-16_genome_1-orf1717	chromosome	1755041	1753470
9		IS66	0730-16_genome_1-orf1718	chromosome	1755408	1755061
9		IS66	0730-16_genome_1-orf1719	chromosome	1756055	1755408
10		IS66	0730-16_genome_1-orf1941	chromosome	1999884	1998313
10		IS66	0730-16_genome_1-orf1942	chromosome	2000251	1999904
10		IS66	0730-16_genome_1-orf1943	chromosome	2000898	2000251
11		IS66	0730-16_genome_1-orf2090	chromosome	2164916	2163345
11		IS66	0730-16_genome_1-orf2091	chromosome	2165283	2164936
11		IS66	0730-16_genome_1-orf2092	chromosome	2165930	2165283
12		IS66	0730-16_genome_1-orf2184	chromosome	2248235	2246664
12		IS66	0730-16_genome_1-orf2185	chromosome	2248602	2248255
12		IS66	0730-16_genome_1-orf2186	chromosome	2249249	2248602
13		IS66	0730-16_genome_1-orf2219	chromosome	2287965	2288450
13		IS66	0730-16_genome_1-orf2219	chromosome	2288450	2288797
13		IS66	0730-16_genome_1-orf2220	chromosome	2288817	2290388
14		IS66	0730-16_genome_1-orf2221	chromosome	2613595	2614242
14		IS66	0730-16_genome_1-orf2506	chromosome	2614242	2614589
14		IS66	0730-16_genome_1-orf2507	chromosome	2614609	2616180
15		IS66	0730-16_genome_1-orf2508	chromosome	2894091	2894381
15		IS66	0730-16_genome_1-orf2803	chromosome	2894411	2894716
15		IS66	0730-16_genome_1-orf2804	chromosome	2894716	2895063
15		IS66	0730-16_genome_1-orf2805	chromosome	2895083	2896654
16		IS66	0730-16_genome_1-orf2806	chromosome	3102068	3100497
16		IS66	0730-16_genome_1-orf2988	chromosome	3102435	3102088
16		IS66	0730-16_genome_1-orf2989	chromosome	3103082	3102435
17		IS66	0730-16_genome_1-orf3199	chromosome	3339044	3337473
17		IS66	0730-16_genome_1-orf3200	chromosome	3339411	3339064

Table S6 IS location in the genome of *E. marmotae* HT073016						
17		IS66	0730-16_genome_1-orf3201	chromosome	3340058	3339411
18		IS66	0730-16_genome_1-orf3213	chromosome	3352617	3351046
18		IS66	0730-16_genome_1-orf3214	chromosome	3352984	3352637
18		IS66	0730-16_genome_1-orf3215	chromosome	3353631	3352984
19		IS66	0730-16_genome_1-orf3832	chromosome	4036253	4036558
19		IS66	0730-16_genome_1-orf3833	chromosome	4036558	4036905
19		IS66	0730-16_genome_1-orf3834	chromosome	4036925	4038496
20		IS66	Contig5-orf10	pEM148	9112	9417
20		IS66	Contig5-orf11	pEM148	9417	9764
20		IS66	Contig5-orf12	pEM148	9784	11355
21		IS66	Contig5-orf141	pEM148	144863	145210
21		IS66	Contig5-orf142	pEM148	145230	146801
21		IS66	Contig5-orf143	pEM148	147386	147847
22		IS66	Contig6-orf36	pEM76	32306	33877
22		IS66	Contig6-orf37	pEM76	33897	34244
22		IS66	Contig6-orf38	pEM78	34244	34891
23		IS66	Contig6-orf52	pEM76	52117	52764
23		IS66	Contig6-orf53	pEM76	52764	53111
23		IS66	Contig6-orf54	pEM78	53131	54702
24		IS66	Contig6-orf58	pEM76	57976	59547
24		IS66	Contig6-orf59	pEM76	59567	59914
24		IS66	Contig6-orf60	pEM78	59914	60561
1		IS3	0730-16_genome_1-orf30	chromosome	30878	31732
1		IS3	0730-16_genome_1-orf490	chromosome	508817	509116
2		IS3	0730-16_genome_1-orf947	chromosome	951199	950345
2		IS3	0730-16_genome_1-orf948	chromosome	951495	951256
3		IS3	0730-16_genome_1-orf1002	chromosome	1008458	1007604
3		IS3	0730-16_genome_1-orf1003	chromosome	1008754	1008455
4		IS3	0730-16_genome_1-orf1164	chromosome	1178073	1177219
4		IS3	0730-16_genome_1-orf1165	chromosome	1178369	1178070
5		IS3	0730-16_genome_1-orf1214	chromosome	1223226	1223525
5		IS3	0730-16_genome_1-orf1215	chromosome	1223522	1224139
6	ISEc16	IS3	0730-16_genome_1-orf2019	chromosome	2081349	2080495
6		IS3	0730-16_genome_1-orf2020	chromosome	2081645	2081346
7		IS3	0730-16_genome_1-orf2510	chromosome	2617142	2617435
7		IS3	0730-16_genome_1-orf2511	chromosome	2617496	2617795
8		IS3	0730-16_genome_1-orf2512	chromosome	2617792	2618646
8		IS3	0730-16_genome_1-orf2513	chromosome	2618685	2619110
9		IS3	0730-16_genome_1-orf3004	chromosome	3117209	3117508
9		IS3	0730-16_genome_1-orf3005	chromosome	3117505	3118359
10		IS3	0730-16_genome_1-orf3848	chromosome	4050638	4050937
10		IS3	0730-16_genome_1-orf3849	chromosome	4050934	4051788
11		IS3	0730-16_genome_1-orf3962	chromosome	4174129	4174428
11		IS3	0730-16_genome_1-orf3963	chromosome	4174425	4175279

Table S6 IS location in the genome of *E. marmotae* HT073016						
12		IS3	0730-16_genome_1-orf4006	chromosome	4227419	4226565
12		IS3	0730-16_genome_1-orf4007	chromosome	4227715	4227416
13		IS3	0730-16_genome_1-orf4276	chromosome	4514006	4514305
13		IS3	0730-16_genome_1-orf4277	chromosome	4514302	4515156
14		IS3	0730-16_genome_1-orf4434	chromosome	4668040	4667186
14		IS3	0730-16_genome_1-orf4435	chromosome	4668336	4668037
15		IS3	Contig5-orf3	pEM148	2374	2673
15		IS3	Contig5-orf4	pEM148	2670	3524
16		IS3	Contig5-orf47	pEM148	43719	44573
16		IS3	Contig5-orf48	pEM148	44570	44869
17		IS3	Contig5-orf50	pEM148	46065	46364
17		IS3	Contig5-orf51	pEM148	46361	47215
18		IS3	Contig6-orf21	pEM76	16748	17602
18		IS3	Contig6-orf22	pEM76	17599	17898
19		IS3	Contig5-orf31	pEM76	27421	27720
19		IS3	Contig6-orf32	pEM76	27717	28571
1	IS1H	IS1	0730-16_genome_1-orf26	chromosome	29126	29398
1		IS1	0730-16_genome_1-orf27	chromosome	29425	29820
2		IS1	Contig5-orf25	pEM148	21981	22271
2		IS1	Contig5-orf26	pEM148	22343	22615
3		IS1	Contig5-orf13	pEM148	11497	11769
1	ISEc1	ISAs1	0730-16_genome_1-orf1720	chromosome	1757255	1756290
1	ISEc13	IS4	0730-16_genome_1-orf293	chromosome	321790	323163
1	IS911	IS3	Contig6-orf1	pEM76	455	757

**Table S7 Pseudogenes with known functions identied in the genome of *E. marmotae* HT073016**				
pseudo gene list	genome locus tag	mutation	Gene	Description
1	0730-16_genome_1-orf34	Stop codon	-	replication protein
	0730-16_genome_1-orf35	Stop codon	-	phage replication protein
2	0730-16_genome_1-orf49	Stop codon	-	phage integrase family protein
	0730-16_genome_1-orf50	Stop codon	-	integrase
3	0730-16_genome_1-orf131	Stop codon	*yfaD*	hypothetical protein
	0730-16_genome_1-orf132	Stop codon	*yfaD*	hypothetical protein
4	0730-16_genome_1-orf143	Stop codon	*yfaL*	adhesin
	0730-16_genome_1-orf144	Stop codon	-	adhesin
	0730-16_genome_1-orf145	Stop codon	*yfaL*	outer membrane autotransporter barrel domain-containing protein
5	0730-16_genome_1-orf207	Stop codon	*setB*	sugar transporter
	0730-16_genome_1-orf208	Stop codon	*yeiO*	sugar transporter
6	0730-16_genome_1-orf261	Stop codon	*molR_C*	molybdate metabolism regulator MolR
	0730-16_genome_1-orf262	Stop codon	-	molybdate metabolism regulator MolR
7	0730-16_genome_1-orf263	Stop codon	-	hypothetical protein
	0730-16_genome_1-orf264	Stop codon	*molR_A*	hypothetical protein
8	0730-16_genome_1-orf299	Stop codon	*alkA*	3-methyl-adenine DNA glycosylase
	0730-16_genome_1-orf300	Stop codon	*alkA*	DNA-3-methyladenine glycosylase 2
9	0730-16_genome_1-orf361	Stop codon	*pduN*	ethanolamine utilization EutN/carboxysome family protein
10	0730-16_genome_1-orf373	Stop codon	*pduF*	propanediol diffusion facilitator
	0730-16_genome_1-orf374	Stop codon	*pduF*	propanediol diffusion facilitator
11	0730-16_genome_1-orf381	Stop codon	*nac*	LysR family transcriptional regulator
	0730-16_genome_1-orf382	Stop codon	*nac*	LysR family transcriptional regulator
12	0730-16_genome_1-orf384	Stop codon	*yeeO*	MATE efflux family protein
	0730-16_genome_1-orf385	Stop codon	*yeeO*	MATE efflux family protein
13	0730-16_genome_1-orf468	Stop codon	*flhA*	flagellar biosynthesis protein flhA
	0730-16_genome_1-orf469	Stop codon	*flhA*	flagellar biosynthesis protein flhA
14	0730-16_genome_1-orf598	Stop codon	*cspC*	Cold shock-like protein cspC
15	0730-16_genome_1-orf626	Stop codon	*yeaL*	membrane protein
	0730-16_genome_1-orf627	Stop codon	*yeaL*	hypothetical protein
16	0730-16_genome_1-orf744	Stop codon	-	inner membrane protein
	0730-16_genome_1-orf745	Stop codon	*ydhK*	inner membrane protein
17	0730-16_genome_1-orf793	Stop codon	*ynfM*	membrane protein
	0730-16_genome_1-orf794	Stop codon	*ynfM*	membrane protein
18	0730-16_genome_1-orf802	Stop codon	*ynfE*	oxidoreductase
	0730-16_genome_1-orf803	Stop codon	*ynfE*	dimethyl sulfoxide reductase subunit A
19	0730-16_genome_1-orf815	Stop codon	-	dicB family protein
	0730-16_genome_1-orf816	Stop codon	-	division inhibition protein DicB
20	0730-16_genome_1-orf878	Stop codon	*fdnG*	formate dehydrogenase, nitrate inducible, alpha subunit, selenocysteine-containing
21	0730-16_genome_1-orf895	Stop codon	-	zinc protease PqqL
	0730-16_genome_1-orf896	Stop codon	-	zinc protease
22	0730-16_genome_1-orf902	Stop codon	-	intimin-like protein SinH
	0730-16_genome_1-orf903	Stop codon	*eaeH*	adhesin/invasin
23	0730-16_genome_1-orf943	Insertion	-	H repeat-associated protein yhhI

Table S7 Pseudogenes with known functions identied in the genome of *E. marmotae* HT073016				
24	0730-16_genome_1-orf953	Stop codon	*yncB*	oxidoreductase, Zn-dependent and NAD-binding
	0730-16_genome_1-orf954	Stop codon	*yncB*	oxidoreductase, Zn-dependent and NAD-binding
25	0730-16_genome_1-orf984	Stop codon	*gapC*	glyceraldehyde-3-phosphate dehydrogenase
	0730-16_genome_1-orf985	Stop codon	*gapC*	glyceraldehyde-3-phosphate dehydrogenase C
26	0730-16_genome_1-orf1001	Insertion	*ompN*	outer membrane porin protein C
	0730-16_genome_1-orf1004	Insertion	*ompN*	outer membrane porin protein C
27	0730-16_genome_1-orf1017	Stop codon	-	hypothetical protein
	0730-16_genome_1-orf1018	Stop codon	-	hypothetical protein
28	0730-16_genome_1-orf1071	Stop codon	*yciQ*	inner membrane protein
	0730-16_genome_1-orf1072	Stop codon	-	inner membrane protein
29	0730-16_genome_1-orf1119	Stop codon	-	fused DNA-binding response regulator NarL and sensory histidine kinase NarX in two-component regulatory system
30	0730-16_genome_1-orf1207	Stop codon	*ybcM*	bacterial regulatory helix-turn-helix s, AraC family protein
31	0730-16_genome_1-orf1284	Stop codon	*flgE*	flagellar hook protein FlgE
	0730-16_genome_1-orf1285	Stop codon	*flgE*	flagellar hook protein FlgE
32	0730-16_genome_1-orf1289	Stop codon	*flgA*	flagellar basal body P-ring biosynthesis protein FlgA
	0730-16_genome_1-orf1290	Stop codon	*flgA*	flagella basal body P-ring formation protein flgA
33	0730-16_genome_1-orf1310	Stop codon	-	multidrug transporter
34	0730-16_genome_1-orf1313	Insertion	*yceK*	lipoprotein
35	0730-16_genome_1-orf1332	Stop codon	*phoH*	hypothetical protein
	0730-16_genome_1-orf1333	Stop codon	*phoH*	protein phoH
36	0730-16_genome_1-orf1586	Stop codon	-	aconitate hydratase 1
	0730-16_genome_1-orf1587	Stop codon	-	aconitate hydratase 1, partial
37	0730-16_genome_1-orf1623	Stop codon	-	glutamate mutase
	0730-16_genome_1-orf1624	Stop codon	*glmL*	hypothetical protein
38	0730-16_genome_1-orf1688	Stop codon	*hutH*	hypothetical protein
39	0730-16_genome_1-orf1721	Stop codon	-	H repeat-associated protein yhhI
40	0730-16_genome_1-orf1724	Stop codon	-	YD repeat
	0730-16_genome_1-orf1725	Stop codon	-	YD repeat
41	0730-16_genome_1-orf1756	Stop codon	*gltJ*	glutamate/aspartate transport system permease gltJ
42	0730-16_genome_1-orf1785	Stop codon	*cspE*	cold shock protein CspE
43	0730-16_genome_1-orf1811	Stop codon	*entB*	2,3-dihydroxybenzoate--AMP ligase
44	0730-16_genome_1-orf1844	Stop codon	*ybcJ*	hypothetical protein
45	0730-16_genome_1-orf1855	Stop codon	*fdrA*	membrane protein
	0730-16_genome_1-orf1856	Stop codon	*fdrA*	protein fdrA
46	0730-16_genome_1-orf1862	Insertion	*allB*	allantoinase
	0730-16_genome_1-orf1865	Insertion	*allB*	allantoinase
47	0730-16_genome_1-orf1872	Stop codon	*ybbS*	LysR family transcriptional regulator
	0730-16_genome_1-orf1873	Stop codon	-	DNA-binding transcriptional activator AllS
58	0730-16_genome_1-orf1889	Stop codon	*siiDA*	hemolysin D
	0730-16_genome_1-orf1890	Stop codon	-	TolC family type I secretion outer membrane protein
49	0730-16_genome_1-orf1896	Insertion	*ushA*	protein UshA
	0730-16_genome_1-orf1897	Insertion	*ushA*	protein UshA
	0730-16_genome_1-orf1901	Insertion	*ushA*	bifunctional UDP-sugar hydrolase/5'-nucleotidase periplasmic precursor
50	0730-16_genome_1-orf1922	Stop codon	*hha*	modulator of gene expression, with H-NS

Table S7 Pseudogenes with known functions identied in the genome of *E. marmotae* HT073016

51	0730-16_genome_1-orf1936	Stop codon	*ybaE*	ABC transporter periplasmic-binding protein
	0730-16_genome_1-orf1937	Stop codon	*ybaE*	hypothetical protein
52	0730-16_genome_1-orf1999	Stop codon	*psiF*	hypothetical protein
53	0730-16_genome_1-orf2003	Stop codon	*yaiZ*	hypothetical protein
54	0730-16_genome_1-orf2010	Insertion	-	membrane protein
	0730-16_genome_1-orf2012	Insertion	-	membrane protein
55	0730-16_genome_1-orf2018	Insertion	*yaiS*	hypothetical protein
	0730-16_genome_1-orf2021	Insertion	*yaiS*	hypothetical protein
56	0730-16_genome_1-orf2037	Stop codon	-	AraC family transcriptional regulator
	0730-16_genome_1-orf2038	Stop codon	-	AraC family transcriptional regulator
57	0730-16_genome_1-orf2044	Stop codon	*codB*	cytosine permease
	0730-16_genome_1-orf2045	Stop codon	*codB*	cytosine permease
58	0730-16_genome_1-orf2053	Stop codon	*yahN*	membrane protein
	0730-16_genome_1-orf2054	Stop codon	-	membrane protein
59	0730-16_genome_1-orf2069	Stop codon	-	AidA-I adhesin-like protein
	0730-16_genome_1-orf2070	Stop codon	*aidA*	outer membrane autotransporter barrel domain-containing protein
60	0730-16_genome_1-orf2142	Stop codon	-	flagellar hook capping protein
	0730-16_genome_1-orf2143	Stop codon	-	flagellar hook capping protein
61	0730-16_genome_1-orf2153	Stop codon	*lfiJ*	hypothetical protein
	0730-16_genome_1-orf2154	Stop codon	*lfiJ*	flagellar export protein FliJ
62	0730-16_genome_1-orf2166	Stop codon	*flhB*	flagellar biosynthesis protein FlhB
	0730-16_genome_1-orf2167	Stop codon	*flhB*	flagellar biosynthesis protein FlhB
63	0730-16_genome_1-orf2168	Stop codon	*lfhA*	flagella biosynthesis protein FlhA
	0730-16_genome_1-orf2169	Stop codon	*fhiA*	flagellar biosynthesis protein FlhA
	0730-16_genome_1-orf2170	Stop codon	-	flagellar biosynthesis protein FlhA
64	0730-16_genome_1-orf2180	Stop codon	-	hypothetical protein
	0730-16_genome_1-orf2181	Stop codon	-	hypothetical protein
65	0730-16_genome_1-orf2280	Stop codon	*hrpB*	ATP-dependent RNA helicase hrpB
	0730-16_genome_1-orf2281	Stop codon	*hrpB*	ATP-dependent RNA helicase hrpB
66	0730-16_genome_1-orf2347	Stop codon	*ftsI*	cell division protein FtsI
	0730-16_genome_1-orf2348	Stop codon	*ftsL*	cell division protein FtsI
67	0730-16_genome_1-orf2473	Stop codon	*hsdR*	type I restriction enzyme EcoKI R protein
	0730-16_genome_1-orf2474	Stop codon	*hsdR*	type I restriction enzyme EcoKI R protein
	0730-16_genome_1-orf2475	Stop codon	*hsdR*	type I restriction enzyme EcoKI subunit R
68	0730-16_genome_1-orf2496	Stop codon	*fimH*	FimH.FliC fusion protein
69	0730-16_genome_1-orf2608	Stop codon	-	dicB family protein
	0730-16_genome_1-orf2609	Stop codon	-	division inhibition protein DicB
70	0730-16_genome_1-orf2682	Stop codon	*rpmJ*	Preprotein translocase subunit secY
71	0730-16_genome_1-orf2701	Stop codon	*rplW*	rpL23-SA fusion protein
72	0730-16_genome_1-orf2719	Stop codon	-	glutathione-regulated potassium-efflux system protein kefB
73	0730-16_genome_1-orf2731	Stop codon	*fic*	cell filamentation protein Fic
74	0730-16_genome_1-orf2751	Stop codon	*dam*	Dam methyltransferase with SUUR CDS
75	0730-16_genome_1-orf2963	Stop codon	*avtA*	valine--pyruvate aminotransferase
	0730-16_genome_1-orf2964	Stop codon	*avtA*	valine--pyruvate aminotransferase

Table S7 Pseudogenes with known functions identied in the genome of *E. marmotae* HT073016				
76	0730-16_genome_1-orf2981	Stop codon	*fdoG*	formate dehydrogenase-O, large subunit homolgoy with fergusonii
77	0730-16_genome_1-orf3060	Stop codon	*rhtC*	threonine transporter RhtB
	0730-16_genome_1-orf3061	Stop codon	*rhtC*	threonine efflux protein
78	0730-16_genome_1-orf3100	Stop codon	*trxA*	thioredoxin/transketolase fusion protein
79	0730-16_genome_1-orf3158	Stop codon	*rpmH*	PREDICTED: ribonuclease P protein component-like
80	0730-16_genome_1-orf3169	Stop codon	-	2-oxo-3-deoxygalactonate 6-phosphate aldolase and galactonate dehydratase
81	0730-16_genome_1-orf3174	Stop codon	*ibpA*	inclusion body protein A - yellow fluorescent protein fusion
82	0730-16_genome_1-orf3217	Insertion	*yicI*	alpha-xylosidase
83	0730-16_genome_1-orf3323	Stop codon	-	transcriptional regulator
	0730-16_genome_1-orf3324	Stop codon	-	transcriptional regulator
84	0730-16_genome_1-orf3325	Stop codon	-	hypothetical protein
	0730-16_genome_1-orf3326	Stop codon	*pgtB*	histidine kinase
85	0730-16_genome_1-orf3398	Stop codon	*yjaB*	acetyltransferase
	0730-16_genome_1-orf3399	Stop codon	*yjaB*	acetyltransferase
86	0730-16_genome_1-orf3474	Stop codon	*yjcO*	hypothetical protein
	0730-16_genome_1-orf3475	Stop codon	*yjcO*	hypothetical protein
87	0730-16_genome_1-orf3482	Stop codon	*fdhF*	formate dehydrogenase H, subunit of formate hydrogenlyase complex
88	0730-16_genome_1-orf3483	Stop codon	*yjcP*	multidrug RND transporter
89	0730-16_genome_1-orf3484	Stop codon	*mdtP*	multidrug RND transporter
90	0730-16_genome_1-orf3488	Stop codon	-	6-phospho-beta-glucosidase
91	0730-16_genome_1-orf3489	Insertion	-	transcription antitermination protein BlgG
	0730-16_genome_1-orf3491	Insertion	-	transcription antitermination protein BlgG
92	0730-16_genome_1-orf3534	Stop codon	-	isovaleryl CoA dehydrogenase
	0730-16_genome_1-orf3535	Stop codon	*aidB*	isovaleryl CoA dehydrogenase
93	0730-16_genome_1-orf3537	Stop codon	*yjfM*	membrane protein
	0730-16_genome_1-orf3538	Stop codon	*yjfM*	hypothetical protein
	0730-16_genome_1-orf3539	Stop codon	*yjfL*	hypothetical protein
94	0730-16_genome_1-orf3602	Insertion	*melB*	sugar:sodium symporter
	0730-16_genome_1-orf3604	Insertion	*melB*	sugar:sodium symporter
95	0730-16_genome_1-orf3617	Stop codon	-	putative alkylphosphonate uptake protein in phosphonate metabolism
96	0730-16_genome_1-orf3670	Stop codon	*ygjJ*	hypothetical protein
	0730-16_genome_1-orf3671	Stop codon	*ygjJ*	hypothetical protein
97	0730-16_genome_1-orf3672	Stop codon	*ygjK*	alpha-glucosidase
	0730-16_genome_1-orf3673	Stop codon	*ygjK*	alpha-glucosidase
98	0730-16_genome_1-orf3686	Stop codon	*yqjA*	inner membrane YqjA domain protein
99	0730-16_genome_1-orf3722	Stop codon	*agaC*	PTS N-acetylgalactosamine transporter subunit IIC
100	0730-16_genome_1-orf3741	Insertion	*yjgN*	membrane protein, partial
	0730-16_genome_1-orf3743	Insertion	*yjgN*	membrane protein
101	0730-16_genome_1-orf3749	Stop codon	-	30S ribosomal protein S21
102	0730-16_genome_1-orf3760	Stop codon	*yqiJ*	inner membrane protein yqiJ
103	0730-16_genome_1-orf3790	Stop codon	-	ABC transporter substrate-binding protein
104	0730-16_genome_1-orf3818	Stop codon	*pitB*	phosphate transporter PitA
	0730-16_genome_1-orf3819	Stop codon	-	inorganic phosphate transporter
105	0730-16_genome_1-orf3839	Stop codon	-	hypothetical protein

**Table S7 Pseudogenes with known functions identied in the genome of *E. marmotae* HT073016**				
	0730-16_genome_1-orf3840	Stop codon	-	type VI secretion protein ImpK
106	0730-16_genome_1-orf3875	Stop codon	*galP*	D-galactose transporter
	0730-16_genome_1-orf3876	Stop codon	*galP*	galactose-proton symporter
107	0730-16_genome_1-orf3944	Stop codon	*ssnA*	protein SsnA
	0730-16_genome_1-orf3945	Stop codon	-	protein SsnA
108	0730-16_genome_1-orf3949	Stop codon	*yqeB*	hypothetical protein
	0730-16_genome_1-orf3950	Stop codon	*yqeB*	hypothetical protein
109	0730-16_genome_1-orf3967	Stop codon	-	bipartite regulatory protein
	0730-16_genome_1-orf3968	Stop codon	*yqeH*	bipartite regulatory protein
110	0730-16_genome_1-orf4076	Stop codon	*ispF*	2-C-methyl-D-erythritol 2,4-cyclodiphosphate synthase
111	0730-16_genome_1-orf4089	Stop codon	*pphB*	serine/threonine-protein phosphatase 2
	0730-16_genome_1-orf4090	Stop codon	*pphB*	serine/threonine-protein phosphatase 2
112	0730-16_genome_1-orf4165	Stop codon	*ygaF*	L-2-hydroxyglutarate oxidase LhgO
	0730-16_genome_1-orf4166	Stop codon	-	L-2-hydroxyglutarate oxidase LhgO
113	0730-16_genome_1-orf4173	Stop codon	*ppnK*	inorganic polyphosphate/ATP-NAD kinase
114	0730-16_genome_1-orf4214	Stop codon	*rnc*	rnc-sfGFP
115	0730-16_genome_1-orf4219	Stop codon	*yfhL*	Sialic acid utilization regulator, RpiR family
116	0730-16_genome_1-orf4233	Stop codon	-	hypothetical protein
	0730-16_genome_1-orf4234	Stop codon	-	hypothetical protein
117	0730-16_genome_1-orf4236	Stop codon	-	fused putative sugar transporter subunits of ABC superfamily: ATP-binding components, partial
	0730-16_genome_1-orf4237	Stop codon	*yphE*	ABC transporter ATP-binding protein
118	0730-16_genome_1-orf4242	Stop codon	*hcaT*	3-phenylpropionic acid MFS transporter
	0730-16_genome_1-orf4243	Stop codon	-	3-phenylpropionic acid MFS transporter
119	0730-16_genome_1-orf4283	Stop codon	*upp*	cytosine deaminase:uracil phosphoribosyltransferase fusion protein
120	0730-16_genome_1-orf4346	Stop codon	-	6-phospho-beta-glucosidase
	0730-16_genome_1-orf4347	Stop codon	-	transcription antitermination protein BlgG
